# *Myeloid Zinc Finger 1*: insights into its oncogenic potential, prognostic value, and impact on immune microenvironment across cancers

**DOI:** 10.3389/fimmu.2025.1591912

**Published:** 2025-06-27

**Authors:** Wenze Tian, Chun Yu, Xin Xu, Jiaqi Li, Shuangfu Peng, Aijun Sun, Shasha Zhang, Chao Jiang

**Affiliations:** ^1^ Department of Thoracic Surgery, The Affiliated Huaian No.1 People’s Hospital of Nanjing Medical University, Huai’an, China; ^2^ General Surgery Department, Lianshui People's Hospital Affiliated to Kangda College of Nanjing Medical University, Huai’an, Jiangsu, China; ^3^ Department of Thyroid and Breast Oncological Surgery, Huai’an Second People’s Hospital, The Affiliated Huaian Hospital of Xuzhou Medical University, Huai’an, Jiangsu, China; ^4^ Faculty of Life Science and Food Engineering, Huaiyin Institute of Technology, Huai’an, Jiangsu, China; ^5^ Key Laboratory of Systems Biomedicine, Shanghai Center for Systems Biomedicine, Engineering Research Center of Techniques and Instruments for Diagnosis and Treatment of Congenital Heart Disease, Institute of Developmental and Regenerative Medicine, Xin Hua Hospital, School of Medicine, Shanghai Jiao Tong University, Shanghai, China; ^6^ Department of Oncology, The Affiliated Huaian No.1 People’s Hospital of Nanjing Medical University, Huai’an, China

**Keywords:** *Myeloid Zinc Finger 1*, pan-cancer, tumor immunity, prognostic biomarker, immunotherapy drug susceptibility

## Abstract

**Background:**

*Myeloid Zinc Finger 1* (*MZF1*) is a zinc finger transcription factor gene that regulates gene expression by recognizing and binding to specific DNA sequences. Preliminary studies have suggested that *MZF1* plays a pivotal role in the invasion and metastasis of various solid cancers. However, its role within the tumor immune microenvironment, as well as its prognostic value and potential for predicting responses to immunotherapy across different cancer types, remains inadequately explored and warrants a comprehensive systematic analysis.

**Methods:**

*MZF1* expression levels in various cancers were obtained from the Cancer Genome Atlas (TCGA) database. The TISCH web tool analyzed *MZF1* expression in 32 cell types. A spatial distribution map of *MZF1* related to cancer tissue markers was created using the STOmics DB. A univariate Cox regression analysis was performed to evaluate *MZF1*’s prognostic value. The cBioPortal database helped explore potential *MZF1* mutations across cancer types. The TIMER2.0 database was used to study the relationship between *MZF1* expression and immune cell infiltration. Gene Set Enrichment Analysis (GSEA) and Gene Set Variation Analysis (GSVA) were performed to elucidate signaling pathways modulated by *MZF1*. Drug sensitivity testing for *MZF1* was done using the CellMiner, the Cancer Therapeutics Response Portal (CTRP), and the Genomics of Drug Sensitivity in Cancer (GDSC) databases. Finally, *MZF1* knockdown was achieved with siRNA silencing.

**Results:**

Changes in *MZF1* expression are linked to the prognosis of most cancer patients. In the tumor microenvironment, *MZF1* is mainly found in CD4 Tconv cells and monocytes/macrophages. Studies show that *MZF1* is associated with cancer immunotherapy markers, immune cell infiltration, and immune modulators. Additionally, its role in immune regulation was confirmed through analysis of StromalScore, ImmuneScore, ESTIMATE, and immune infiltration. Molecular docking identified *MZF1*-targeted drugs, with validated effects on breast cancer and gastric cancer cell survival and migration *in vitro*. Lastly, the knockdown of *MZF1* can suppress cancer cell migration.

**Conclusion:**

Collectively, these findings underscore the pivotal role of *MZF1* in tumor biology and immune modulation. *MZF1* emerges as a promising prognostic biomarker and potential therapeutic target, offering novel avenues for cancer treatment strategies.

## 1 Introduction

Cancer remains the foremost cause of morbidity and mortality in numerous countries worldwide, posing a significant threat to both human lifespan and public health (1). The Global Cancer Statistics 2022 report offers a comprehensive overview of incidence and mortality data for 36 cancer types across 185 countries. Notably, breast cancer ranked as the second most common malignancy among women globally in 2022, accounting for 11.6% of all cancer cases (2–4). In recent years, cancer immunotherapy has emerged as a highly promising therapeutic strategy. However, a substantial proportion of patients fail to derive satisfactory clinical benefit from immune checkpoint blockade therapies. Consequently, a comprehensive understanding of the molecular underpinnings of cancer, along with the identification of novel biomarkers and therapeutic targets, is essential for advancing cancer diagnosis and treatment.

With ongoing technological advances, numerous computational tools have been developed to analyze large-scale gene expression datasets, such as those provided by The Cancer Genome Atlas (TCGA), with the aim of quantifying individual gene expression or inferring the abundance of specific cell populations from transcriptomic profiles. The Gene Signature–based TCGA (GS-TCGA) platform further enables researchers to perform survival analyses linked to gene expression signatures, investigate the functional relevance of gene co-expression patterns, and uncover putative gene regulatory mechanisms. These approaches offer valuable insights into the molecular pathogenesis of cancer and patient prognosis, providing a stronger scientific foundation for the development of more effective strategies for cancer prevention and therapy (5).

*Myeloid Zinc Finger 1* (*MZF1*) is a member of the zinc finger transcription factor family, belonging to a subfamily of zinc finger proteins (6). It was initially isolated from the peripheral blood of patients with chronic myelogenous leukemia, where it plays a pivotal role in the transcriptional regulation of hematopoietic development (7). Subsequent studies have demonstrated that *MZF1* regulates the expression of genes crucial for cellular differentiation, proliferation, and programmed cell death, with aberrant expression potentially contributing to the onset of hematologic malignancies (8). Further research has established a clear association between *MZF1* and the progression of various solid tumors, where it facilitates the growth, migration, and invasion of cancer cells in breast cancer, liver cancer, lung adenocarcinoma, gastric cancer, and others (9–13). In the early stages of research, *MZF1* was primarily studied for its role in hematopoietic differentiation and leukemia (14). In the context of leukemia, *MZF1* was found to inhibit the differentiation of hematopoietic stem cells by suppressing the activity of the CD34 or c-myb promoters, thereby impeding differentiation and facilitating the emergence of a leukemia-like phenotype (15). In gastric cancer, *MZF1* interacts with HMGB3 to regulate the expression of target genes, thereby influencing the proliferation, metastasis, and invasion of gastric cancer cells. Some studies have suggested that *MZF1* expression correlates with poor prognosis in gastric cancer (16). As research into *MZF1* deepens, it has become clear that both its underexpression and overexpression are linked to cancer progression. As a multifunctional transcription factor, the molecular mechanisms underlying *MZF1*’s actions remain an active area of study. Its role in the tumor immune microenvironment, along with its prognostic and predictive value for immunotherapy responses across different cancer types, has yet to be fully understood. Therefore, a deeper understanding of the pathological mechanisms involving *MZF1* and the identification of potential therapeutic targets are crucial directions for future research.

This study aimed to comprehensively analyze MZF1 expression and its mechanistic roles across various cancer types. The findings of this study could help develop new therapeutic strategies for advancing cancer research and clinical interventions.

## 2 Methods

### 2.1 Public data and processing

Initially, the *MZF1* gene was queried using the OpenTargets platform (URL: https://platform.opentargets.org/), and the top 35 diseases most strongly associated with *MZF1*—ranked by Europe PMC relevance—were selected for visualization. Additionally, the Human Protein Atlas (HPA) database (URL: https://www.proteinatlas.org/) provided detailed information regarding the subcellular localization of the *MZF1* protein. Next, immunohistochemistry (IHC) was employed to observe the differential expression of *MZF1* in normal tissues and cancerous tissues, such as stomach adenocarcinoma and breast cancer. Subsequently, RNA sequencing data from the TIMER database were utilized to analyze *MZF1* expression across various tumor types. The Toil pipeline was employed to efficiently process large-scale data in a unified manner, ensuring consistency of results while minimizing costs (17). Furthermore, the Cancer Cell Line Encyclopedia (CCLE) (URL: https://portals.broadinstitute.org/ccle/) provided data from 33 different cancer cell lines, which included *MZF1* expression levels, facilitating the assessment of *MZF1* expression in various cancer cell models. Moreover, the GeneMANIA tool (http://www.genemania.org) was used to construct a protein-protein interaction (PPI) network, exploring proteins that interacted with or were co-expressed with *MZF1* (18). This tool also offered information regarding *MZF1*’s involvement in physical interactions, immune-related pathways, predictions, co-localization, genetic interactions, and shared protein domains (19). Additionally, the GSE99254 and GSE120575 datasets (URL: https://www.ncbi.nlm.nih.gov/geo/) were analyzed. The GSE99254 dataset included single-cell RNA sequencing data of 12,346 T cells isolated from the peripheral blood of 14 non-small cell lung cancer (NSCLC) patients, while the GSE120575 dataset comprised data from 16,291 immune cells derived from 48 melanoma (SKCM) patients treated with checkpoint inhibitors. Detailed information on cancer abbreviations can be found in **Supplementary Table S1**. This study was approved by the Ethics Committee of the research institution (Approval No. HYIT-EC-2024-012).

### 2.2 Single-cell and spatial localization analysis of *MZF1*

Using the TISCH network tool (20), the expression levels of *MZF1* across 32 distinct cell types were analyzed. This analysis was conducted from three perspectives: *MZF1* gene expression, cell type annotations (primarily based on lineage), and cancer types. The expression levels of *MZF1* within different cell types were quantitatively assessed and visualized through heatmaps, scatter plots, and violin plots. Additionally, spatial transcriptomic data obtained from the STOmics DB database (https://db.cngb.org/stomics/) (21) were utilized to analyze the spatial similarities within the tumor microenvironment, which provided further insights into the spatial distribution and context of *MZF1* expression.

### 2.3 Survival prognosis analysis of *MZF1* in pan-cancer patients

Using the TCGA database and the PanCanSurvPlot platform (https://smuonco.shinyapps.io/PanCanSurvPlot/), a COX regression analysis was conducted to assess the expression levels of *MZF1* across various cancer types and its impact on the survival prognosis of pan-cancer patients. The analysis focused on four key prognostic indicators: overall survival (OS), disease-specific survival (DSS), disease-free interval (DFI), and progression-free survival (PFS). Subsequently, optimal cut-off values for pan-cancer classification were determined using the Illumina HiSeq platform. Hazard ratios (HR) were calculated using the R package, and the results were visualized using forest plots, with a 95% confidence interval (95% CI).

### 2.4 Mutation and genomic alterations of *MZF1* in cancer

Genomic alterations of the *MZF1* gene across various cancer types were analyzed using the cBioPortal database (https://www.cbioportal.org/) (22). Subsequently, the GSCA database (http://bioinfo.life.hust.edu.cn/GSCA) (23) was employed to investigate the methylation differences of *MZF1* in cancer tissues, as well as the correlation between its mRNA expression levels and methylation status. The samples were categorized into three groups—WT, Amp, and Dele—and survival rate differences related to *MZF1* gene alterations and copy number variations (CNV) were assessed across pan-cancer. Additionally, Spearman’s correlation analysis was performed to evaluate the relationship between *MZF1* methylation and mRNA expression. Tumor samples were stratified into high and low methylation groups to further analyze survival disparities. To minimize statistical variance, time-to-event analysis was conducted to assess differences in OS, DSS, and PFS.

### 2.5 Predictive analysis of immunotherapy

Somatic mutation data obtained from the TCGA database (https://tcga.xenahubs.net) were analyzed using the R package “maftools,” enabling the calculation of TMB and MSI for each TCGA cancer sample. Spearman’s rank correlation coefficient was then employed to evaluate the relationship between *MZF1* expression and both TMB and MSI. The results were subsequently visualized using radar plots generated by the “ggradar” package in R. The immune therapy outcomes were categorized into four groups: progressive disease (PD), stable disease (SD), complete remission (CR), and partial remission (PR). Utilizing the “survminer” R package, the optimal cutoff point was determined to divide the immune therapy cohorts into low *MZF1* and high *MZF1* groups, where the survival rates and therapeutic responses of each group were assessed. Additionally, the expression of MMR (mismatch repair) genes—namely MLH1, MSH2, MSH6, PMS2, and EPCAM—across various cancer types was examined to explore their correlation with *MZF1* expression. Finally, heatmaps generated using the “tidyverse” and “ggnewscale” R packages were employed to illustrate the findings.

### 2.6 Relationship between *MZF1* expression and immune factors

The expression of *MZF1* and its correlation with the TME were analyzed based on Illumina platform data. Subsequently, the R packages “limma” and “ESTIMATE” were employed to comprehensively evaluate the stromal score, immune score, and ESTIMATE score across different tumor types, to further explore their relationships. To investigate the association between *MZF1* expression and cancer immune response, Spearman’s correlation analysis was conducted using data from the TCGA and TIMER 2.0 databases. This analysis focused on the correlation between *MZF1* expression and immune cell infiltration, as well as immune response-related genes, across various cancer types. Furthermore, immune-related genes were downloaded from the TISDB database, including 150 genes associated with MHC, immune suppressive factors, chemokine receptors, immune activators, and chemokine proteins (24), and their relationship with *MZF1* was analyzed.

### 2.7 Biological significance of *MZF1*

In order to further explore the biological implications of *MZF1*, Gene Set Enrichment Analysis (GSEA) and Gene Set Variation Analysis (GSVA) were performed. These analyses were carried out utilizing a suite of R packages, including “tidyverse,” “limma,” “org.Hs.eg.db,” “gseaplot2,” and “clusterProfiler” (25). The C2 and C5 gene sets were sourced from the classification database. Normalized enrichment scores were computed, followed by a comparison of the *MZF1* low-expression and high-expression groups, which were categorized according to distinct disease classifications. The false discovery rate (FDR) was determined based on *MZF1* expression levels, with an intermediate threshold employed to stratify the samples into high and low expression cohorts, thereby generating GSVA scores for all disease-related pathways. Furthermore, additional R packages, including “GSVA,” “ggprism,” “GSEABase,” “ggthemes,” “BiocAllegal,” and “clusterProfiler,” were also employed for the GSVA analysis.

### 2.8 Susceptibility of *MZF1* to relevant drugs and molecular docking analysis

The relationship between *MZF1* expression and its response to various pharmacological agents, as well as the degree of drug sensitivity, was investigated using the CellMiner platform (URL: http://discover.nci.nih.gov/cellminer/) (26), the CTRP database (URL: http://portals.broadinstitute.org/ctrp/), and the GDSC database (URL: https://www.cancerrxgene.org/). These platforms were also used to compile drug screening data from the NCI-60 cancer cell line panel. Data from FDA-approved and clinical trial-stage drugs were analyzed using the R package “limma,” with data containing missing values exceeding 80% being excluded. The remaining missing data were imputed using the “Impute” package in R. The data were visualized using the “ggplot2” and “ggpubr” packages, with statistical significance set at a *p-value* of < 0.05. Subsequently, molecular docking analyses were performed using Autodock4 (27) to assess the binding affinity between *MZF1* and Panobinostat. The molecular structure of Panobinostat, obtained from the PubChem database (URL: https://pubchem.ncbi.nlm.nih.gov/), and the predicted 3D structure of *MZF1*, provided by AlphaFold (URL: https://alphafold.ebi.ac.uk/) (28), served as the basis for the docking studies. Finally, the docking models were visualized using PyMOL software for further analysis and exploration.

### 2.9 Cell culture and transfection procedures

The human breast cancer cell line MCF-7 and the gastric cancer cell line HGC-27 were obtained from the Cell Bank of the Chinese Academy of Sciences, Shanghai, China. Cells were cultured in media supplemented with 10% fetal bovine serum (FBS; Procell) and maintained under standard conditions of 37°C and 5% CO2. Mycoplasma contamination was monitored regularly using PCR-based methods. For gene silencing experiments, siRNAs targeting *MZF1* and its corresponding negative control were procured from GenePharma (Shanghai, China). According to the manufacturer’s instructions, the siRNAs were transfected into cells using the Lipofectamine 3000 reagent (Invitrogen, California, USA). The specific sequences of the siRNAs employed are provided in **Supplementary Table S2**.

### 2.10 Reverse transcription quantitative polymerase chain reaction (RT-qPCR) protocol

Total RNA was extracted using TRIzol reagent (Takara Bio, Kusatsu, Japan), and RNA concentration and purity were assessed using the NanoDrop 2000 system (Thermo Scientific). Reverse transcription was performed with PrimeScript™ RT Premix (Takara, RR036A), followed by quantitative PCR using SYBR^®^ Premix Ex Taq™ II (Takara, RR820A). Gene expression levels were quantified using the 2-ΔΔCt method, with ACTB serving as the internal reference. The primer sequences for RT-qPCR are provided in **Supplementary Table S3**.

### 2.11 Cell viability assay

Cell viability was assessed using the CCK-8 reagent kit (GK10001, GLPBIO, Montclair, California, USA). Cells were seeded in a 96-well plate at a density of 2 × 10³ per well and incubated for 24 or 48 hours. Following incubation, 10 μl of CCK-8 solution was added to each well, and the cells were further incubated for 2 hours. The absorbance at 450 nm was measured using a microplate reader, and the results were subsequently analyzed.

### 2.12 Colony formation assay

For the colony formation assay, the MCF-7 and HGC-27 cell lines were subjected to separate experiments, with 1,000 cells per well seeded in 6-well plates. Each condition was performed in triplicate. Fresh culture medium was replenished every three days. After one week of incubation, the cells were fixed with methanol and subsequently stained with 0.5% crystal violet. Finally, colonies were captured and quantified using ImageJ software.

### 2.13 Cell scratch wound healing assay

For the wound healing assay, cells transfected with the designated siRNA were seeded in a 6-well plate at a density of 1 × 10^5^ cells per well, with the culture medium supplemented with 2% fetal bovine serum to prevent cell proliferation from affecting the results. A scratch was introduced into the monolayer using a micropipette tip. Images of the scratched area were then captured using a Nikon Ti-E inverted microscope (Nikon Instruments, Florence, Italy) at 0 and 48 hours. Subsequently, the wound area at each time point was measured and analyzed using ImageJ software, with the area at each time point normalized to the area at T0.

### 2.14 Statistical analysis

Upon completion of the experiments, bioinformatics validation analysis was performed. Initially, datasets were curated by removing missing values and duplicates. The TPM values were then transformed using log2(TPM + 1). To compare the expression of *MZF1* between normal and tumor tissues, the Mann-Whitney U test (Wilcoxon rank-sum test) was applied. Expression levels of *MZF1* based on data from the CCLE database were analyzed using the Kruskal-Wallis test. Finally, depending on whether the samples were paired, paired or unpaired t-tests were employed to compare *MZF1* gene expression between different groups or between tumor and normal tissues. Statistical significance was set at *p-values* < 0.05. All statistical analyses were conducted using R software (version 4.4.0, https://www.R-project.org).

## 3 Results

### 3.1 Differential expression of *MZF1* in pan-cancer

**Figure 1:**
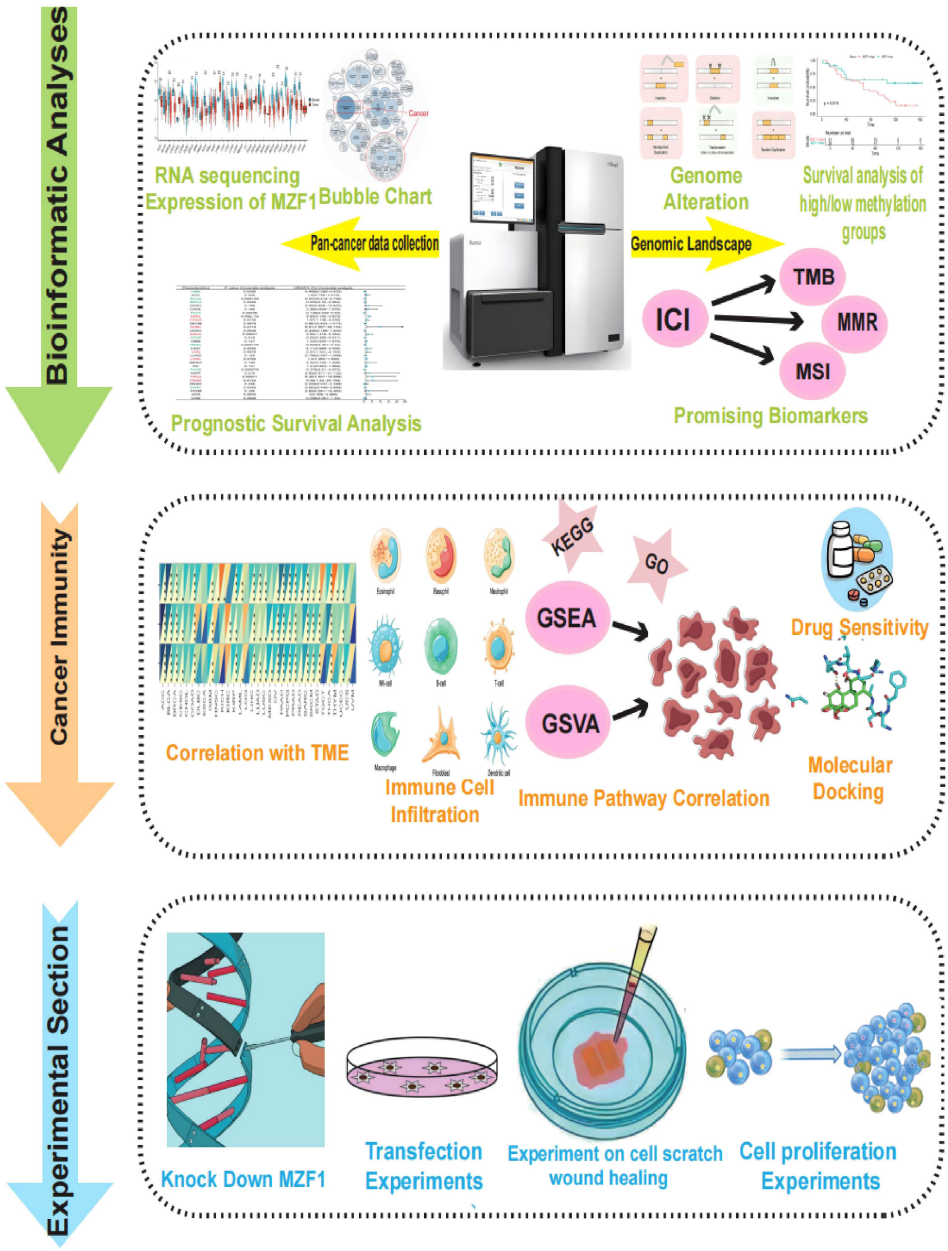
The study’s methodological framework delineates a comprehensive investigation of the target gene *MZF1* across a broad spectrum of cancers. Initially, the expression of *MZF1* in pan-cancer was evaluated, followed by an exploration of its associations with various cancer subtypes. The analysis compared *MZF1* expression disparities across 33 malignant and non-malignant tissues, alongside distinct cellular environments. Subsequent investigations incorporated single-cell transcriptomic analyses and prognostic evaluations. A thorough examination of the genomic landscape was performed, emphasizing genomic instability, with data sourced from the cBioPortal and GSCA databases to assess pan-cancer alterations, including CNVs and DNA methylation patterns. Further, the correlation between *MZF1* expression and factors such as TMB, MSI, and MMR status was explored. To unravel the functional role of *MZF1* in carcinogenesis, the study investigated its involvement in immune modulation, evaluating sequence function enrichment, immune checkpoint regulation, cytokine receptor interactions, and immune cell infiltration. Lastly, potential therapeutic implications of *MZF1* were assessed through predictions of chemotherapy responses, drug sensitivity profiles, and relevant experimental evaluations. *MZF1*, *Myeloid Zinc Finger 1*; GSCA, the Gene Set Cancer Analysis; CNVs, copy number variations; TMB, tumour mutational burden; MSI, microsatellite instability; MMR, mismatch repair.

**Figure 2:**
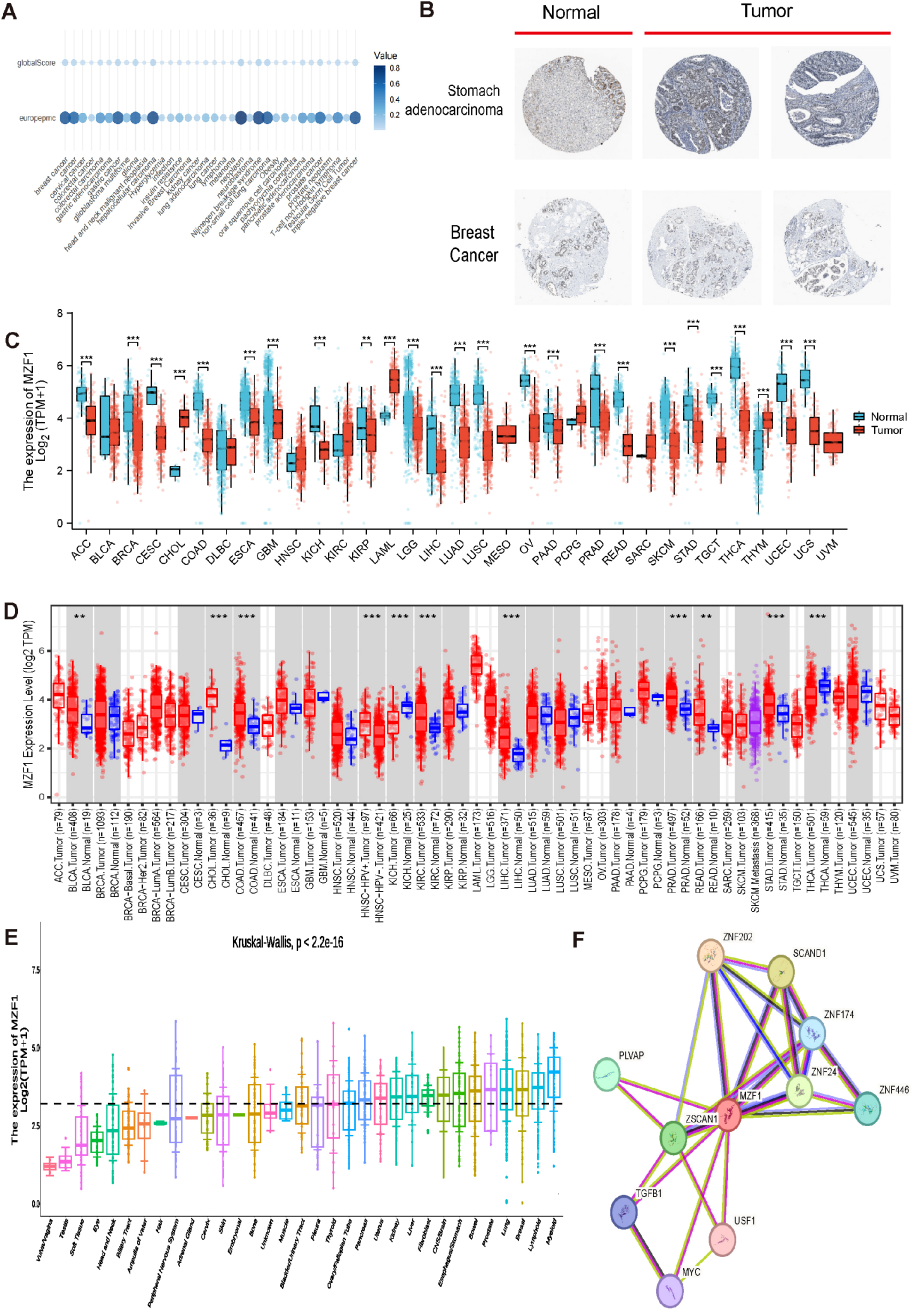
Differential expression and associated genes of *MZF1* in pan-cancer. **(A)** Expression of *MZF1* in different cancers. **(B)** Immunohistochemical analysis of *MZF1* protein expression in normal (left) versus tumor (right) tissues. **(C)** Pan-cancer expression data from the TIMER database were utilized to assess the expression differences of *MZF1*, based on RNA sequencing results. Statistical significance markers were *p-values < 0.05,**p-values < 0.01 ***p-values < 0.001. **(D)** *MZF1* mRNA expression levels in pan-cancer and corresponding control tissues were analyzed via TIMER 2.0. (Statistical significance was marked as *p-values < 0.05, **p-values < 0.01, ***p-values < 0.001) **(E)** The Kruskal-Wallis test was employed to evaluate the differences in *MZF1* expression across 33 distinct organs. **(F)** PPI network analysis was conducted to identify potential *MZF1* binding partners. *MZF1*, *Myeloid Zinc Finger 1*; PPI, protein-protein interaction.

**Figure 1** illustrates the overall scope of the study. Initially, the top 35 diseases most closely associated with *MZF1* were selected using the OpenTargets platform, based on relevance scores ranked by Europe PMC (29). The resulting visualization revealed that the cancers most strongly associated with *MZF1* included breast cancer, gastric cancer, hepatocellular carcinoma, neoplasm, Nijmegen breakage syndrome, non-small cell lung carcinoma, prostate cancer, and triple-negative breast cancer (**Figure 2A**). Subsequently, IHC was utilized to examine the differential expression of *MZF1* protein in gastric adenocarcinoma, breast cancer, and normal tissues. As shown in **Figure 2B**, *MZF1* expression was significantly elevated in gastric adenocarcinoma compared to normal tissues. RNA sequencing data were further analyzed via the TIMER database to explore the expression of *MZF1* across various cancer types. The results indicate a pronounced upregulation of *MZF1* in CHOL, LAML, THYM, PCPG, and HNSC, while a notable downregulation was observed in ACC, BRCA, CESC, COAD, LUAD, LUSC, OV, READ, TGCT, THCA, UCEC, and USC (**Figure 2C**). Further validation through TIMER 2.0 reaffirmed these observations, highlighting the mRNA expression discrepancies between tumor and normal tissues. Notably, *MZF1* expression was markedly elevated in the majority of tumor types, particularly in CHOL, LIHC, BLCA, COAD, KIRC, PRAD, and PEAD (**Figure 2D**). Analysis of samples derived from various organs provided deeper insight into the expression patterns of *MZF1* across different tissue types. The results revealed that *MZF1* expression was significantly higher in specific organs, such as the liver, stomach, lungs, and breast (**Figure 2E**). The STRING tool was employed to construct a protein-protein interaction network for *MZF1*, elucidating its interactions and co-expression with other proteins. The analysis identified the top ten proteins with the strongest interactions with *MZF1*, including ZNF202, SCAND1, ZNF174, PLVAP, ZNF24, USF1, ZNF446, ZSCAN1, TGFB1, and MYC (**Figure 2F**). These proteins are likely to play pivotal roles in the initiation and progression of cancer.

### 3.2 Single-cell analysis of *MZF1* expression

**Figure 3:**
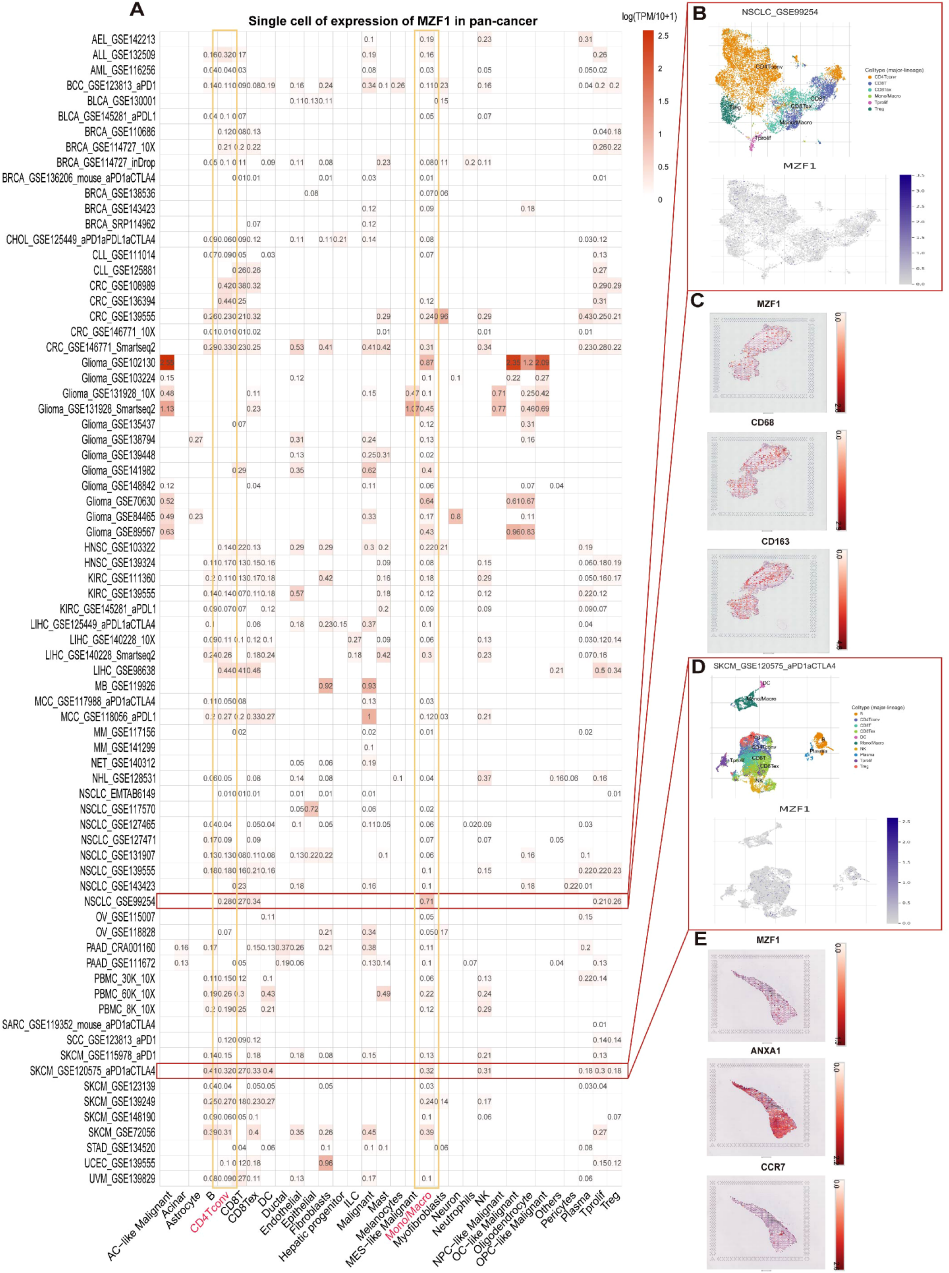
Single-cell analysis of *MZF1* in various cancers. **(A)** Overview of *MZF1* expression across 32 cell types derived from 77 single-cell datasets. **(B)** Scatter plot depicting the distribution of 6 distinct cell types in the GSE99254 NSCLC dataset, along with *MZF1* expression levels for each cell type. **(C)** Spatial transcriptomic slices illustrating the localized expression of *MZF1*, CD68, and CD163 markers, with dot color reflecting the expression intensity of these markers. **(D)** Scatter plot showing the distribution of 10 different cell types in the GSE120575 SKCM dataset, with *MZF1* expression levels displayed for each cell type. **(E)** Spatial transcriptomic slices demonstrating the spatial distribution of *MZF1*, *ANXA1*, and *CCR7* markers, where dot color corresponds to the expression level of the markers. *MZF1*, *Myeloid Zinc Finger 1*; *ANXA1*, *Annexin A1*; *CCR7*, *C-C Chemokine Receptor 7*; NSCLC, non-small cell lung cancer.

To elucidate the predominant cell types associated with *MZF1* in the TME, a single-cell dataset of 75 cancer samples was processed using single-cell analysis. Utilizing the TISCH network tool, the expression levels of *MZF1* were examined across 32 distinct cell types, encompassing immune cells, stromal cells, tumor cells, and functional cells (20). The results revealed that *MZF1* is predominantly expressed in immune cells, particularly in CD4 Tconv cells and monocytes/macrophages (**Figure 3A**). Furthermore, analysis of the GSE99254 dataset, which includes single-cell data from 12,346 T cells derived from the peripheral blood of 14 NSCLC patients undergoing treatment, demonstrated that *MZF1* is primarily expressed, albeit at lower levels, in CD4 Tconv cells, CD8 Tex cells, CD8 T cells, and monocytes/macrophages within the NSCLC microenvironment (**Figure 3B**). Additionally, spatial transcriptomic data from the STOmics DB database was leveraged to investigate the spatial distribution of *MZF1*. Notably, a significant spatial overlap was observed between *MZF1* and the M2 macrophage markers CD163 and CD68 in NSCLC tumor tissue (**Figure 3C**). In the GSE120575 dataset, which includes tumor samples from 48 SKCM patients treated with checkpoint inhibitors, *MZF1* expression was found predominantly in CD4 Tconv cells, Treg cells, CD8 T cells, and CD8 Tex cells within the SKCM microenvironment (**Figure 3D**). Further spatial similarity analysis via the STOmics DB database revealed that *MZF1* exhibited significant spatial co-localization with tumor cell marker ANXA1 and T cell marker *C-C Chemokine Receptor 7* (*CCR7*) within SKCM tumor tissue, suggesting potential co-expression of *MZF1*, *Annexin A1* (*ANXA1*), and *CCR7* in these cell types (**Figure 3E**).

### 3.3 Expression levels of *MZF1* and prognostic analysis in pan-cancer

**Figure 4:**
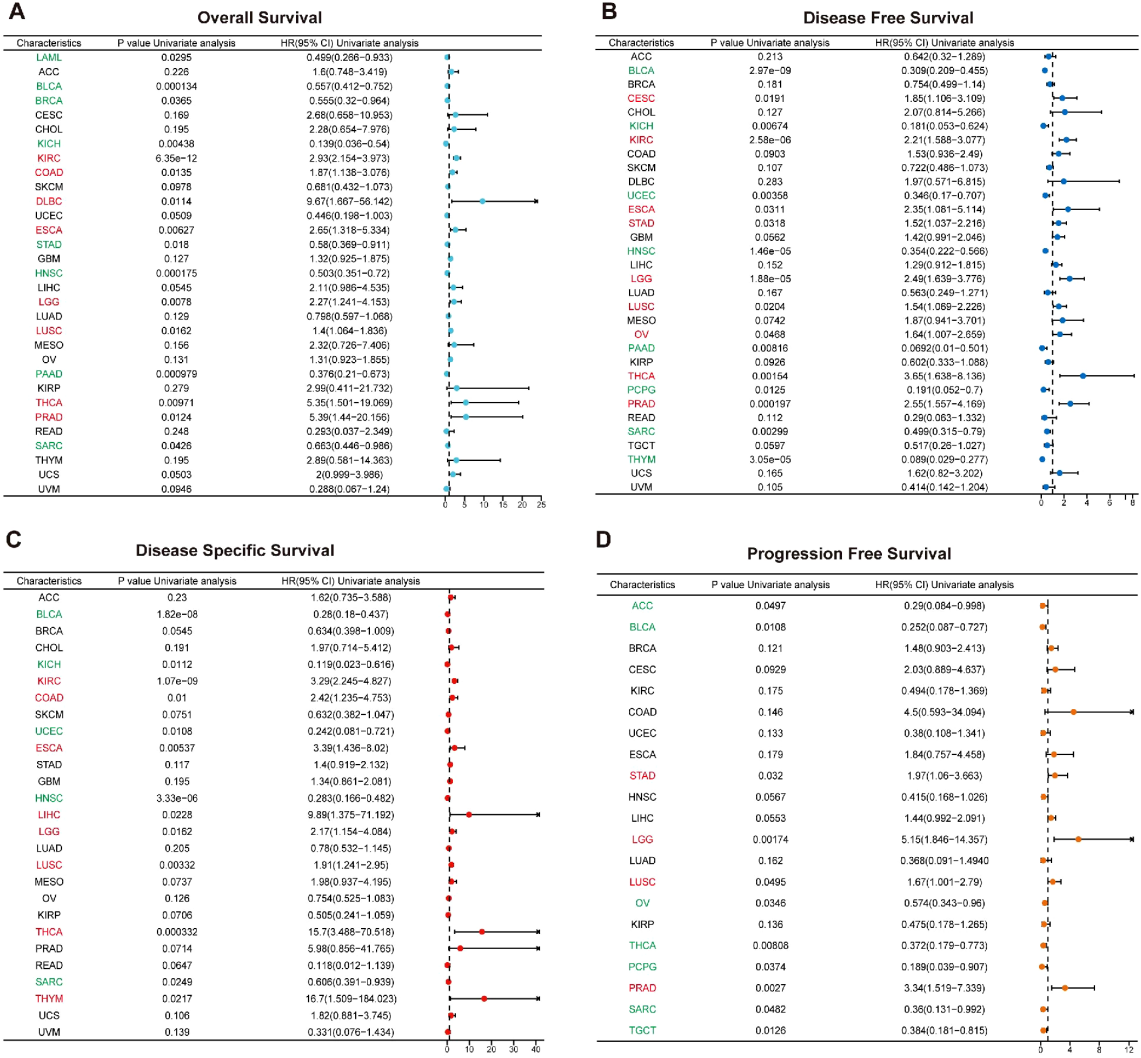
Prognostic role of *MZF1* expression in pan-cancer. The Forest plot illustrates the prognostic significance of *MZF1* expression across various cancer types, derived from univariate Cox regression analysis. *MZF1* expression was found to correlate with OS **(A)**, DFS **(B)**, DSS **(C)**, and PFS **(D)**. Cancer types marked in red indicate that *MZF1* acts as a statistically significant risk factor (HR > 1), while those in green signify that *MZF1* serves as a statistically significant protective factor (HR < 1). *MZF1*, *Myeloid Zinc Finger 1*; OS, overall survival; DSS, disease-specific survival; DFI, disease-free interval; PFS, progression-free survival.

To assess the prognostic significance of *MZF1* across various cancer types, a COX regression analysis was performed using data from the TCGA database to examine the expression levels of *MZF1* in different cancer cohorts (30). The prognostic analysis in breast cancer revealed that *MZF1* expression is significantly associated with the prognosis of multiple cancer types. Further investigation utilizing the COX proportional hazards model elucidated the role of *MZF1* in diverse malignancies. Regarding OS, the analysis indicated that *MZF1* acts as a protective factor in patients with LAML, BLCA, BRCA, KICH, STAD, HNSC, PAAD, and SARC, while it serves as a risk factor in those with KIRC, COAD, DLBC, ESCA, LGG, LUSC, THCA, and PRAD (**Figure 4A**). Additionally, DFS analysis revealed that *MZF1* functions as a risk factor in CESC, KIRC, ESCA, STAD, LGG, LUSC, OV, THCA, and PRAD, whereas it acts as a protective factor in BLCA, KICH, UCEC, HNSC, PAAD, PCPG, SARC, and THYM (**Figure 4B**). Given that OS outcomes often encompass non-cancer-related mortality, which may introduce confounding factors, conducted a more precise analysis using DSS to better correlate survival outcomes with effective cancer therapies. The results indicated that *MZF1* acts as a protective factor in BLCA, KICH, UCEC, HNSC, and SARC, while serving as a risk factor in KIRC, COAD, ESCA, LIHC, LGG, LUSC, THCA, and THYM (**Figure 4C**). Finally, in the PFS analysis, *MZF1* was found to be a risk factor in STAD, LGG, LUSC, and PRAD, while it functioned as a protective factor in ACC, BLCA, OV, THCA, PCPG, SARC, and TGCT patients (**Figure 4D**).

### 3.4 Alterations in the *MZF1* gene and its association with genomic instability in cancer

**Figure 5:**
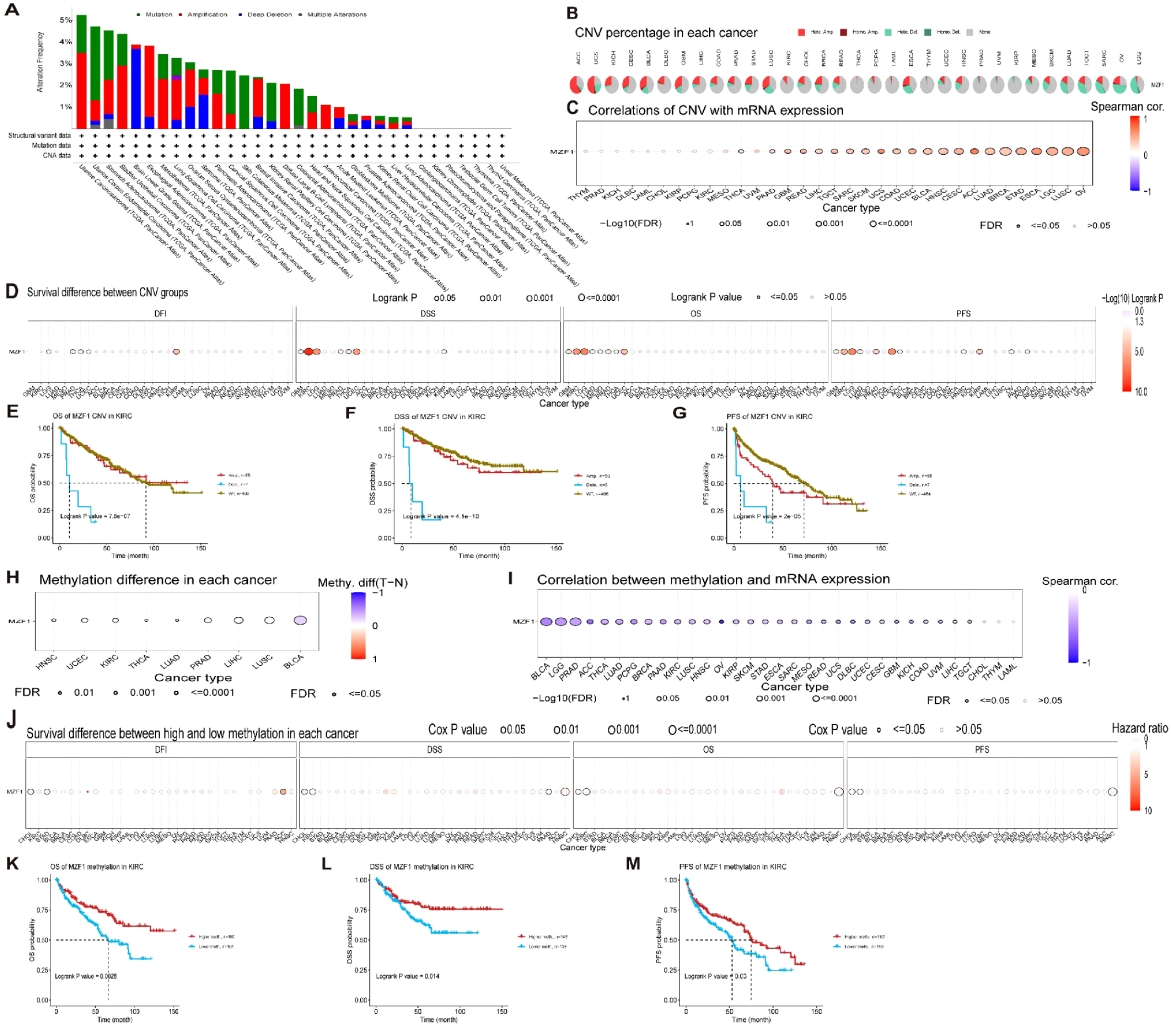
*MZF1* correlation with genomic instability in TCGA tumors. **(A)** Genomic alterations of *MZF1*, including mutations, amplifications, deep deletions, and various other modifications, were examined within the TCGA pan-cancer cohort. **(B)** A summary of CNVs of the *MZF1* gene across different cancer types is presented. **(C)** The relationship between *MZF1* CNV and mRNA expression was assessed through Spearman’s correlation analysis. **(D)** Correlation between *MZF1* CNV status and clinical outcomes, including OS, DSS, PFS, and DFI, was explored. **(E-G)** Kaplan-Meier survival curves were generated using the GSCA web tool to evaluate the prognostic significance of *MZF1* CNVs in patients with KIRC. **(H)** Methylation differences were analyzed in cancers with more than 10 tumor-normal sample pairs, with only results showing *p-values ≤*0.05 being displayed. **(I)** Spearman’s correlation analysis was conducted to explore the relationship between *MZF1* methylation and mRNA expression. **(J)** The association between *MZF1* methylation status and clinical outcomes, including OS, DSS, PFS, and DFI, was analyzed. **(K-M)** Kaplan-Meier survival curves were utilized to assess survival outcomes in KIRC patients based on high and low *MZF1* methylation status. *MZF1*, *Myeloid Zinc Finger 1*; TCGA, the Cancer Genome Atlas; CNVs, copy number variations; OS, overall survival; DSS, disease-specific survival; DFI, disease-free interval; DFI, PFS, progression-free survival.

The onset of cancer is intricately linked to genetic mutations, which disrupt the normal regulation of cellular growth and division, thereby precipitating tumor formation (31). To explore the genomic alterations of the *MZF1* gene across various cancer types, conducted an analysis using the cBioPortal database. Our findings revealed that among TCGA datasets, the most prevalent alteration of the *MZF1* gene was observed in Uterine Carcinosarcoma, with alteration frequencies exceeding 5%. Notably, the majority of these alterations were amplifications, with a smaller proportion resulting in mutations (**Figure 5A**). Further analysis focused on the CNV and DNA methylation of the *MZF1* gene. Using the GSCA database, first examined the percentage of CNV across 33 cancer types, which revealed that the frequency of heterozygous amplification was markedly higher than that of heterozygous deletions (**Figure 5B**). Then investigated the correlation between *MZF1* CNV and mRNA expression. The results demonstrated a significant positive correlation between *MZF1* CNV and mRNA expression across most cancer types, with particularly strong correlations observed in UCS, ACC, ESCA, and OV (**Figure 5C**). Additionally, survival analysis indicated that *MZF1* CNV is associated with lower OS in certain cancer types, including GBM, KIRC, LGG, UCEC, and KIRP (**Figure 5D**). To further assess the impact of *MZF1* CNV on survival, stratified the samples into WT, Amp, and Dele groups, and compared survival outcomes across these groups. In KIRC patients, no statistically significant difference in survival was observed between the WT and Amp groups (**Figures 5E-G**). DNA methylation, a common epigenetic modification, can inactivate tumor suppressor genes and contribute to carcinogenesis (32). Next, examined the methylation differences across various cancer samples and found that tumor samples in BLCA, LUSC, LIHC, and PRAD exhibited significantly higher methylation levels than normal tissues, whereas tumors in HNSC, UCEC, and KIRC showed lower methylation levels (**Figure 5H**). In addition, a Spearman correlation analysis was performed to explore the relationship between *MZF1* gene methylation and mRNA expression. The results indicated a strong association between *MZF1* methylation and mRNA expression in most cancer types, with particularly pronounced correlations in BLCA, LGG, PRAD, ACC, and OV (**Figure 5I**). Further stratification of tumor samples based on methylation levels into high-methylation and low-methylation groups revealed survival differences across cancer types. Elevated methylation in CHOL and KIRC was associated with poorer survival, whereas higher methylation in HNSC was linked to improved survival outcomes (**Figure 5J**). Lastly, a deeper investigation revealed that high levels of *MZF1* methylation were associated with a reduced risk of mortality in KIRC patients (**Figures 5K-M**).

### 3.5 Association between *MZF1* gene expression, immune modulators, TMB, and MSI

**Figure 6:**
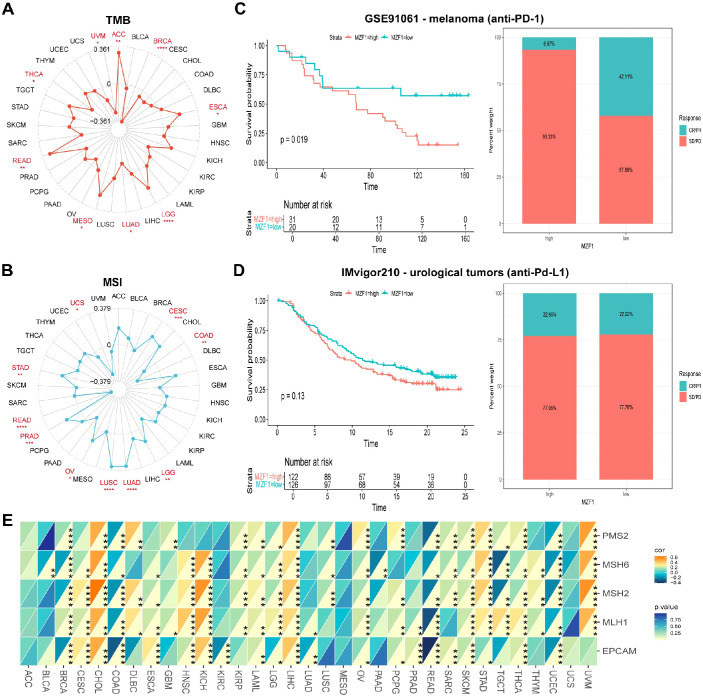
Association between *MZF1* expression and TMB, MSI, and MMR across different cancer types. Radar plots were employed to visualize the correlation between *MZF1* expression and tumor mutational burden (TMB) **(A)** and microsatellite instability (MSI) **(B)**. **(C, D)** Kaplan-Meier survival curves and stacked bar graphs illustrating survival outcomes and immunotherapy response proportions in low and high *MZF1* expression patient groups from two independent cohorts: GSE91061 (top) and IMvigor210 (bottom). **(E)** A heatmap demonstrates the correlation between *MZF1* expression and five mismatch repair (MMR) genes in a pan-cancer cohort. Statistical significance is indicated as follows: *p-values < 0.05, **p-values < 0.01, ***p-values < 0.001, and ****p-values < 0.0001. *MZF1*, *Myeloid Zinc Finger 1*; TMB, tumor mutational burden; MSI, microsatellite instability.

To explore the role of the *MZF1* gene in predicting the efficacy of ICIs, further investigated the correlation between *MZF1* expression and two key biomarkers of ICI sensitivity: TMB and MSI. Both TMB and MSI have been established as crucial biomarkers for evaluating ICI responsiveness, with significant implications for patient prognosis and therapeutic outcomes (33, 34). TMB, defined as the total number of mutations within a tumor’s genome, serves as a measure of tumor immunogenicity and is a promising predictor of response to immunotherapy in cancer patients (35, 36). MSI, an extreme mutation pattern at microsatellite loci, results from defects in the MMR system and is commonly observed in cancers such as colorectal, endometrial, and gastric adenocarcinomas (37). The MMR status has been recognized as a vital predictor of ICI response (38). In our analysis of the correlation between *MZF1* expression and TMB, significant positive correlations were observed in ACC, ESCA, LGG, MESO, and READ, whereas negative correlations were evident in BRCA, LUAD, THCA, and UVM (**Figure 6A**). Regarding MSI, *MZF1* expression was positively correlated with MSI in CESC, LGG, LUAD, LUSC, OV, PRAD, and STAD, while it showed negative correlations in COAD, PEAD, and UCS (**Figure 6B**). These findings suggest that *MZF1* holds potential as a predictor for the efficacy of ICIs across different cancer types. Subsequently, stratified patients into two groups: those treated with anti-PD-1 therapy and those receiving anti-PD-L1 therapy, to assess the predictive role of *MZF1* expression in these cohorts. In the GSE91061 melanoma cohort, found that *MZF1* expression was negatively correlated with response to anti-PD-1 treatment, with patients exhibiting lower *MZF1* expression showing significantly better survival outcomes than those with higher *MZF1* expression (**Figure 6C**). Conversely, in the IMvigor210 urothelial carcinoma cohort, *MZF1* expression was positively correlated with response to anti-PD-L1 treatment, with high *MZF1* expression associated with improved survival and extended overall survival compared to lower expression levels (**Figure 6D**). Also examined the relationship between *MZF1* expression and the expression levels of key MMR genes, including MLH1, MSH2, MSH6, PMS2, and EPCAM (**Figure 6E**). In 33 cancer types, *MZF1* expression was significantly negatively correlated with the expression of MMR genes in BLCA, BRCA, COAD, KIRC, LUAD, LUSC, READ, and UCEC, while a significant positive correlation was observed in CESC, CHOL, HNSC, KICH, LIHC, STAD, and UVM. In conclusion, the expression of *MZF1* emerges as a significant predictor of immune treatment response, highlighting its potential as a valuable biomarker in immunotherapy. Notably, *MZF1* could play an instrumental role in the predictive framework for immune therapeutic responses across diverse cancer types.

### 3.6 Association between *MZF1* gene expression and immune-related factors

**Figure 7:**
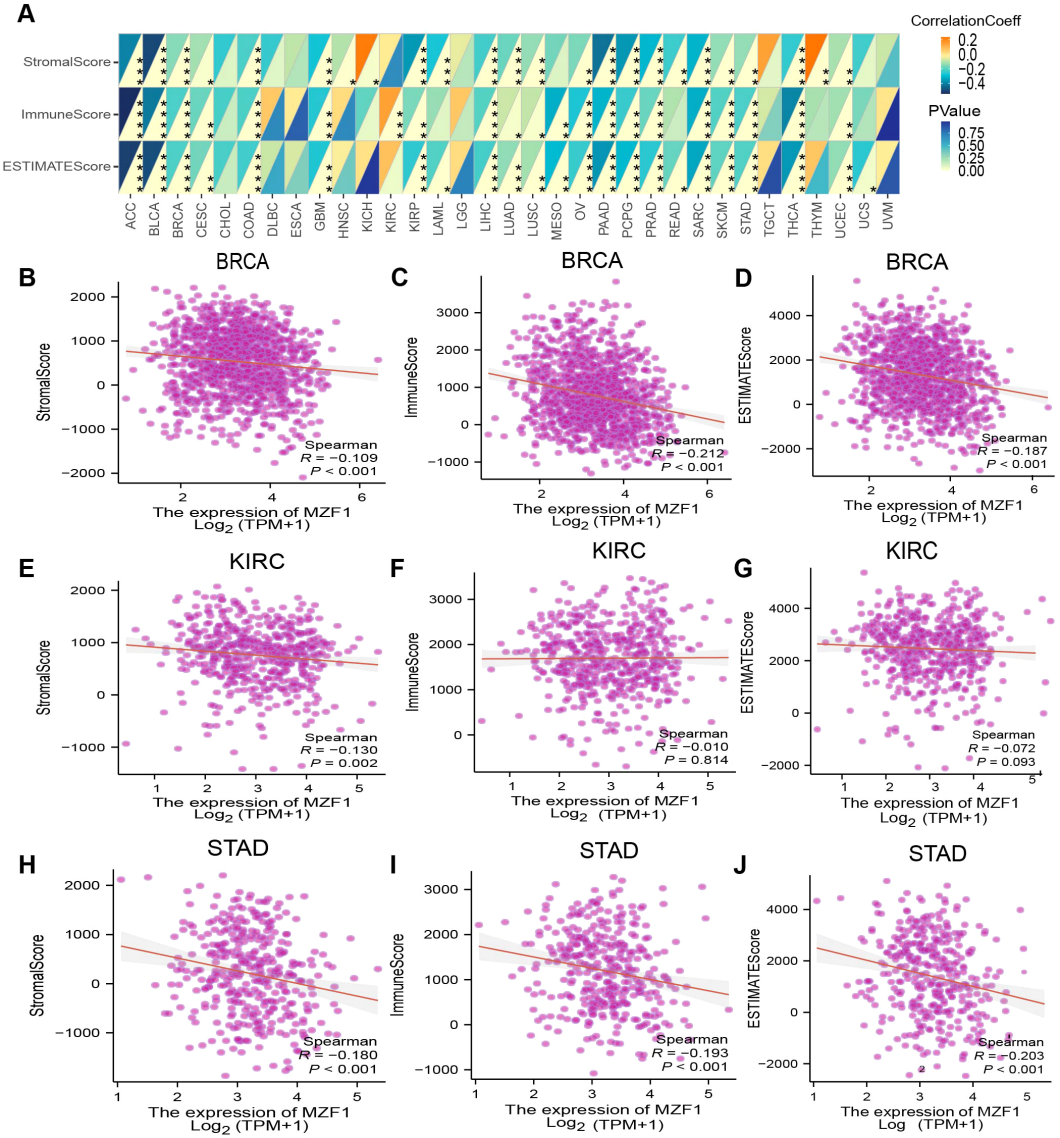
Analysis of *MZF1* expression and immune characteristics. **(A)** The relationships between *MZF1* expression and ImmuneScore, StromalScore, and EstimateScore were assessed through Spearman’s correlation analysis. The correlation coefficients are presented in the upper-left triangle, while *p-value*s are shown in the lower-right triangle (*p-values < 0.05, **p-values < 0.01, ***p-values < 0.001, ****p-values < 0.0001). The relationship between *MZF1* expression and these three scores in specific cancer types is as follows: ACC **(B-D)**, BRCA **(E-G)**, and UCEC **(H-J)**. *MZF1*, *Myeloid Zinc Finger 1*.

To enhance therapeutic outcomes for cancer patients, a comprehensive understanding of the TME is indispensable. The TME is a complex ecosystem that supports the survival and progression of tumor cells, characterized by intricate interactions between cancer cells and various components of the surrounding microenvironment. These interactions play a pivotal role in tumor progression and the development of resistance to anti-cancer therapies. The TME comprises both cellular and non-cellular elements, with the cellular components including tumor-associated macrophages, cancer-associated fibroblasts, endothelial cells, and NK cells, among others (39). In this study, we further explored the relationship between *MZF1* gene expression and the TME. By evaluating the stromal index, immune index, and ESTIMATE scores across various tumor types, found that *MZF1* expression was generally negatively correlated with the TME in most cancer types. However, in certain cancers such as KICH, KIRC, and THYM, a significant positive correlation between *MZF1* expression and the TME was observed (**Figure 7A**). Specifically, the relationship between *MZF1* and tumor types such as BRCA, KIRC, and STAD is illustrated in **Figures 7B–J**.

### 3.7 Correlation between *MZF1* gene expression, immune cell infiltration, and immune regulatory genes

**Figure 8:**
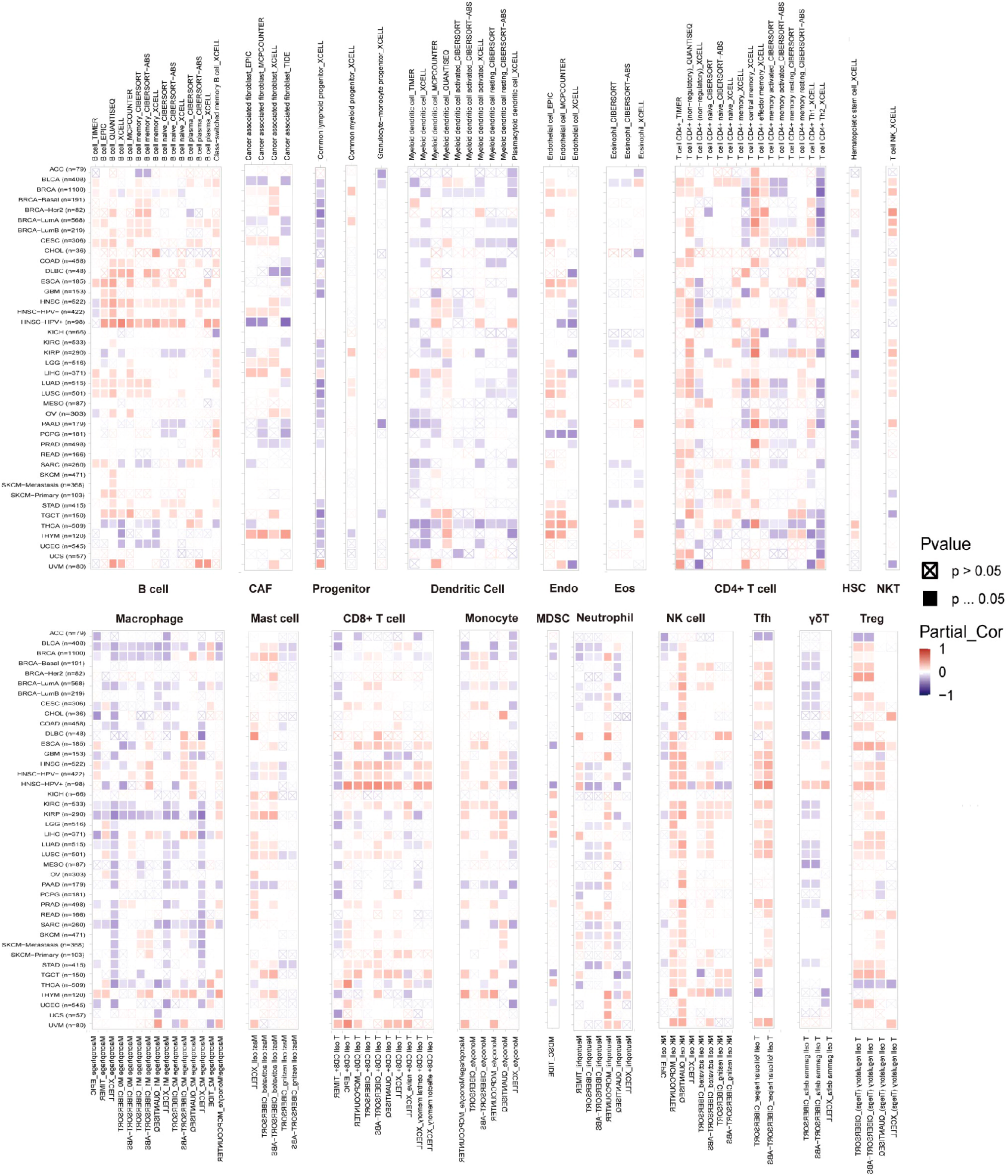
*MZF1* gene TIMER analysis of immune cell infiltration. The expression of *MZF1* in pan-cancer with B cells, CAF cells, endothelial cells, eosinophils, CD4+ T cells, NKT cells, CD8+ T cells, mast cells, monocytes, and Tregs cells were analyzed from the TIMER2.0 database. Red is positively correlated, and blue is negatively correlated. *MZF1*, *Myeloid Zinc Finger 1*; CAFs, cancer-associated fibroblasts; NKT cells, natural killer T.

**Figure 9:**
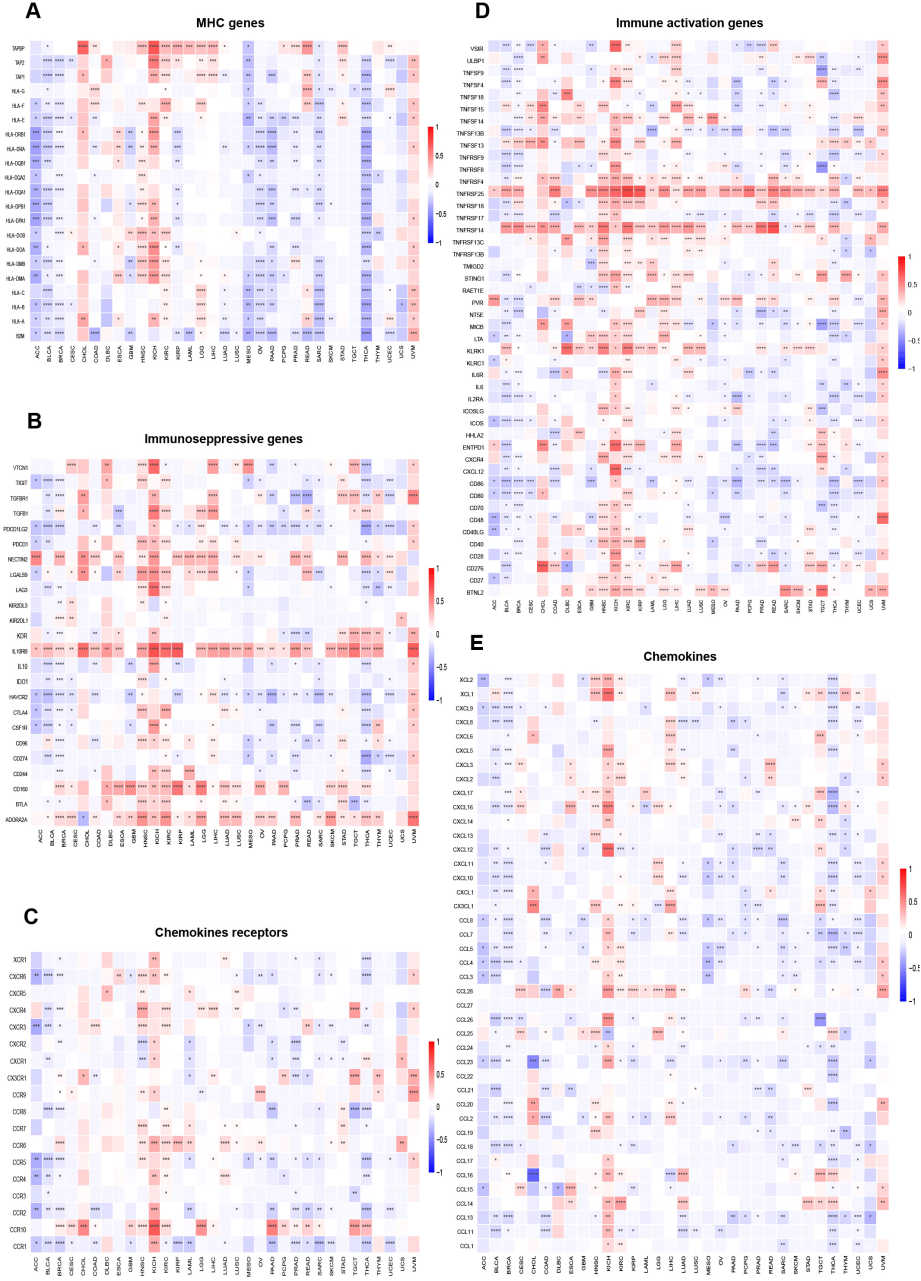
Relationship between *MZF1* expression and immune-related factors. The correlation between *MZF1* expression and various immune-related components was examined, including: **(A)** Chemokine receptors, **(B)** Chemokines, **(C)** Immunosuppressive factors, **(D)** Immunostimulatory factors, and **(E)** MHC genes. Statistical significance was indicated as *p-values < 0.05, **p-values < 0.01, ***p-values < 0.001, and ****p-values < 0.0001. (*Myeloid Zinc Finger 1* (*MZF1*), major histocompatibility complex (MHC)).

The link between immune cell infiltration and *MZF1* expression was intensively investigated to determine the interrelationship between *MZF1* and tumour immunity. Spearman correlation analysis was conducted to examine the correlation between *MZF1* gene expression and immune cell infiltration across various cancer types. The cell types investigated included B cells, cancer-associated fibroblasts (CAF), progenitor cells, dendritic cells, endothelial cells, eosinophils, CD4+ T cells, hematopoietic stem cells (HSC), natural killer T (NKT) cells, CD8+ T cells, macrophages, mast cells, monocytes, MDSC, neutrophils, NK cells, Tfh cells, γ/δ T cells, and Tregs cells. The analysis revealed that *MZF1* expression was significantly positively correlated with B cells, CAF cells, endothelial cells, eosinophils, CD4+ T cells, NKT cells, CD8+ T cells, mast cells, monocytes, and Tregs cells in most TCGA tumor types. In contrast, a significant negative correlation was observed between *MZF1* and progenitor cells, dendritic cells, macrophages, HSC cells, MDSC cells, and γ/δ T cells (**Figure 8**). To further investigate the relationship between *MZF1* gene expression and immune response genes, analyzed the correlation between *MZF1* and key immune regulatory genes. These genes primarily encode major histocompatibility complex (MHC) proteins, immune checkpoint molecules, immune activation proteins, chemokine receptors, and various chemokines. The results indicated that *MZF1* expression was significantly negatively correlated with immune response genes in most cancer types. However, a notable positive correlation was observed in CHOL, ESCA, HNSC, KICH, KIRC, LGG, LIHC, and UVM (**Figure 9**).

### 3.8 Biological significance of *MZF1* gene expression across cancers

**Figure 10:**
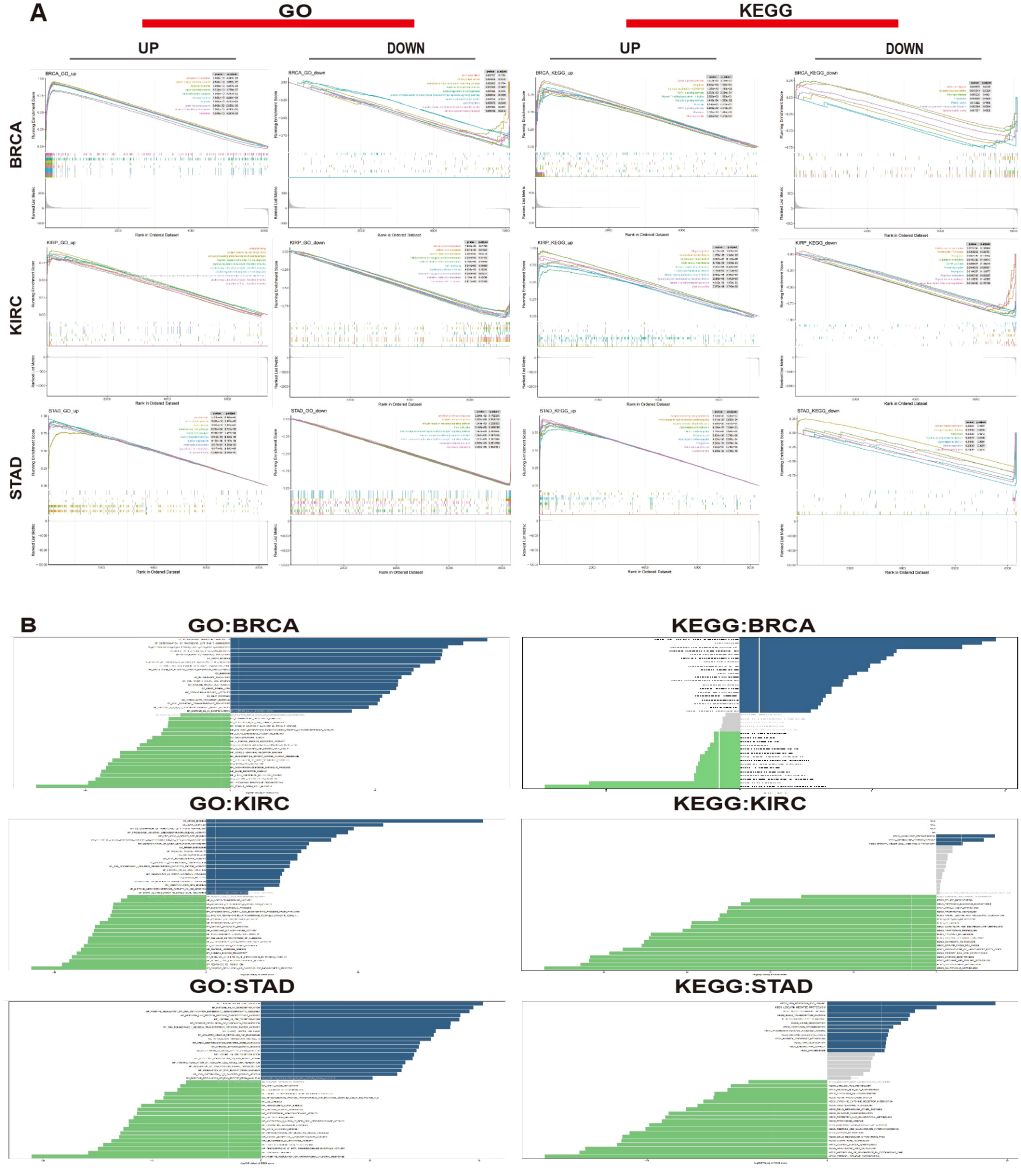
Biological significance of *MZF1* in tumors. **(A)** The GO functional annotation and KEGG pathway analysis demonstrate the GSEA results for *MZF1* in BRCA, KIRC, and STAD. The different colored curves represent regulatory functions in various cancer types. Peaks on the ascending curve indicate positive regulation, while those on the descending curve indicate negative regulation. **(B)** GSVA analysis using GO and KEGG datasets was performed for BRCA, KIRC, and STAD. The blue bars represent pathways with positive correlations, green bars represent pathways with negative correlations, and gray bars indicate pathways with no significant correlation (FDR > 0.05). The horizontal axis shows the log10 (*P-value*) of the GSVA score. *MZF1*, *Myeloid Zinc Finger 1*; GO, Gene Ontology; KEGG, Kyoto Encyclopedia of Genes and Genomes; GSEA, the Gene Set Enrichment Analysis; GSVA, Gene Set Variation Analysis; FDR, the false discovery rate.

To investigate the role of *MZF1* in various cancers, sequence functional enrichment analysis tools, such as GSEA and GSVA, were employed to explore its biological significance across different cancer types. By analyzing the median expression levels of *MZF1* across various cancers, the samples were classified into high-expression and low-expression groups, and GSEA and GSVA were performed to uncover the bioinformatics enrichment patterns associated with this gene. In the GSEA enrichment analysis, KEGG (Kyoto Encyclopedia of Genes and Genomes) and GO (Gene Ontology) methods were applied (40), yielding the following results: In BRCA, *MZF1* positively regulated pathways such as cytoplasmic translation, cytosolic large ribosomal subunit, cytosolic ribosome, ribonucleoprotein complex, ribosome, and Coronavirus disease − COVID−19. However, *MZF1* showed negative regulation in pathways like the isoprenoid biosynthetic process, regulation of endoplasmic reticulum stress-induced intrinsic apoptotic signaling pathway, Parkinson’s disease, and protein export. In KIRC, *MZF1* acted as a positive regulator in antigen binding, negative regulation of lymphocyte-mediated immunity, antigen processing and presentation of peptide antigens, graft-versus-host disease, and autoimmune thyroid disease. Conversely, it displayed negative regulation in processes such as mineral absorption, porphyrin metabolism, cellular transition metal ion homeostasis, and transition metal ion transport. In STAD, *MZF1* was a negative regulator in glycerolipid metabolism, oxidative phosphorylation, and antigen receptor-mediated signaling pathways, but positively regulated pathways like Vibrio cholerae infection, viral myocarditis, and monoamine transport (**Figure 10A**). These results suggest that *MZF1* plays a positive regulatory role in immune-related activities in BRCA, KIRC, and STAD. To further elucidate the biological significance of *MZF1* in tumors, GSVA analysis was conducted, because of its robustness (41). The results revealed that *MZF1* showed positive correlations with most immune-related activities, including immune transport, immune surveillance, immune rhythm, immune response, inflammatory response, DNA repair mechanisms, and histone methylation. However, it exhibited negative correlations with immune activities such as reproductive immunity, immune responses, biosynthesis, glycolysis, immune-related responses, regulation of Treg cell function, immune cell survival and proliferation, immune response regulation, and antimicrobial tumor responses (**Figure 10B**).

### 3.9 Sensitivity of *MZF1* to related drugs and molecular docking analysis

**Figure 11:**
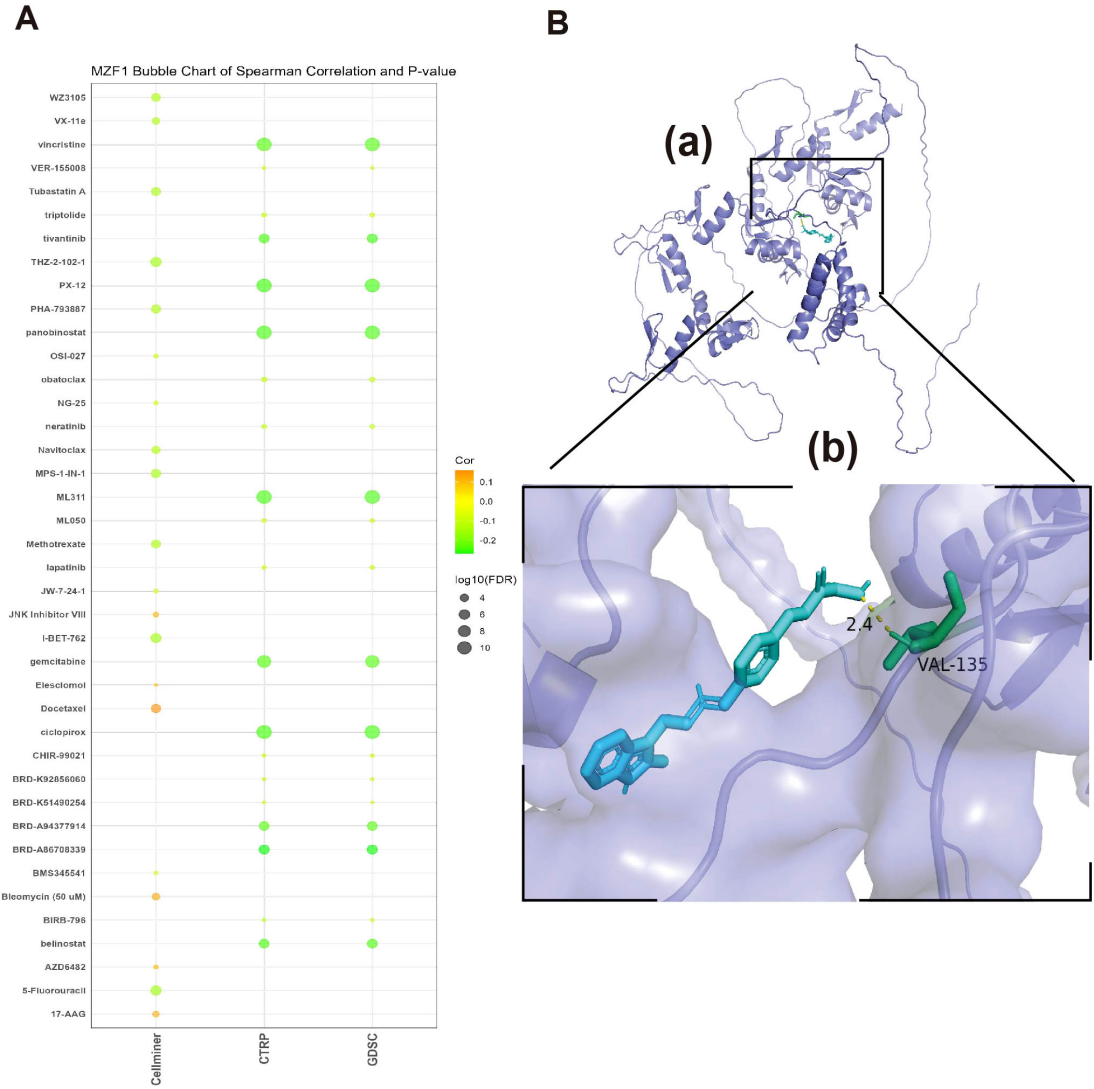
Relationship between *MZF1* and drug susceptibility and molecular docking analysis of targeted compounds **(A)** The association between *MZF1* expression and predicted drug responses was analyzed using data from the CellMiner, CTRP, and GDSC databases. **(B)** A computational prediction of the interaction between Panobinostat and the *MZF1* protein was performed. (a) The schematic representation of the *MZF1* protein’s band structure and the molecular structure of Panobinostat are depicted. (b) An enlarged view illustrates the interaction between Panobinostat and the *MZF1* protein. *MZF1*, Myeloid Zinc Finger 1; CTRP, the Cancer Therapeutics Response Portal; GDSC, the Genomics of Drug Sensitivity in Cancer.

In this study, to assess the correlation between *MZF1* and various drugs, a sensitivity analysis was conducted. The results indicated that *MZF1* expression exhibited significant correlations with the sensitivity of multiple drugs. For instance, drugs such as JNK Inhibitor VIII, Elesclomol, Docetaxel, Bleomycin, AZD6482, and 17−AAG showed a positive correlation with *MZF1* expression, suggesting that *MZF1* may enhance the anti-cancer efficacy of these drugs. On the other hand, some drugs, including Vincristine, Tivantinib, PX−12, Panobinostat, ML311, Gemcitabine, Ciclopirox, BRD−A86708339, Belinostat, BRD−A94377914, 5−Fluorouracil, I−BET−762, Methotrexate, Navitoclax, MPS−1−IN−1, Tubastatin A, WZ3105, and VX−11e, displayed significant negative correlations with *MZF1* expression (**Figure 11A**). These findings suggest that *MZF1* may influence the anti-tumor effects of these drugs through various mechanisms across different cancer types. To verify the accuracy of these findings, molecular docking analysis was further conducted using Autodock4 to assess the binding affinity between *MZF1* protein and anti-cancer drugs. Molecular docking analysis provides a clear visualization of the binding sites of the drug and *MZF1* protein, along with the maximum binding energy. In the presented results, “Panobinostat (LBH589)”, a novel HDAC inhibitor, has been shown to inhibit breast cancer progression by exosome-mediated suppression and demonstrate anti-tumor activity at lower concentrations (42, 43). Recent studies suggest that Panobinostat can inhibit EGFR expression in EGFR-mutant lung cancer cells, and the combination of EGFR-TKI drugs like Erlotinib with Panobinostat may synergistically suppress lung cancer cell proliferation (44). Additionally, Panobinostat has shown potential in combination with Trastuzumab for the treatment of HER2-positive metastatic breast cancer and demonstrated therapeutic potential against aggressive triple-negative breast cancer cells (45, 46). The molecular docking analysis revealed that *MZF1* and Panobinostat form a stable binding structure through hydrogen bonds, with the maximum binding energy of -4.28 kJ/mol (**Figure 11B**). These findings highlight the potential role of *MZF1* in drug response, particularly its interaction with drugs like Panobinostat. This suggests that *MZF1* could serve as a valuable drug target or prognostic biomarker, aiding in predicting drug efficacy and optimizing anti-cancer treatment regimens.

### 3.10 Relationship between *MZF1* expression silencing and cell proliferation

**Figure 12:**
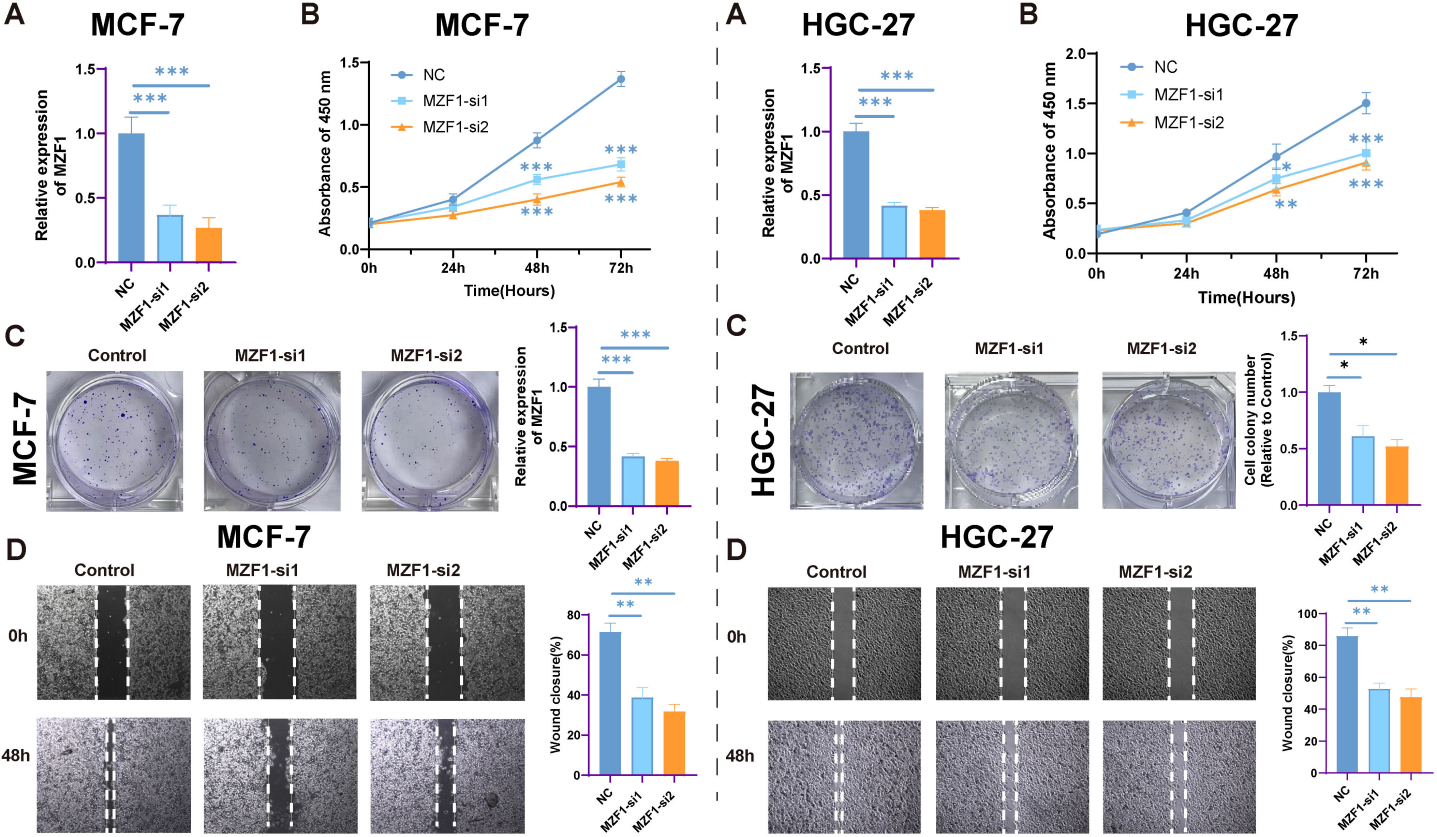
The impact of *MZF1* knockdown on cell proliferation. **(A)** The relative expression levels of *MZF1* in the NC, *MZF1*-si1, and *MZF1*-si2 groups post knockdown (Statistical significance was marked as ***p-values < 0.001). **(B)** CCK-8 assay results indicate that *MZF1* knockdown significantly inhibits the proliferation of breast cancer cells. (Statistical significance was marked as ***p-values < 0.001). **(C)** Comparison of colony formation abilities between the reference cell line and the *MZF1*-si1 and *MZF1*-si2 groups (Statistical significance was marked as **p-values < 0.01). **(D)** Wound healing assays were conducted on cancer cells from the NC, *MZF1*-si1, and *MZF1*-si2 groups to evaluate migratory potential (mark to *p-values < 0.05, **p-values < 0.01). *MZF1*, Myeloid Zinc Finger 1; NC, the negative control.

In this study, the relationship between *MZF1* expression levels and cancer cell proliferation was investigated by silencing *MZF1* expression using siRNA. Cancer cell lines were divided into three groups: NC, *MZF1*-si1, and *MZF1*-si2, to explore the role of *MZF1*. The experimental results showed that as *MZF1* expression was reduced, the proliferation capacity of the cells was significantly limited. Specifically, in the three experimental groups, the expression of *MZF1* decreased over time. It was observed that, under the same optical density, the expression level of *MZF1* showed a continuous decrease as time progressed (**Figures 12A, B**). This finding suggests that the expression level of *MZF1* is closely associated with the proliferative status of the cells. Furthermore, when *MZF1* expression was silenced, a significant reduction in the proliferation of breast cancer cells was observed. These cells exhibited lower proliferative capacity during culture, indicating the critical role of *MZF1* in tumor cell proliferation(**Figure 12C**). The experiment also assessed the impact of reduced *MZF1* expression on cancer cell migration. The results showed that silencing *MZF1* expression led to a significant decrease in the migration ability of cancer cells (**Figure 12D**). These findings suggest that *MZF1*’s high expression not only influences the proliferation of cancer cells but may also play a crucial role in cell migration and metastasis. In conclusion, *MZF1* expression levels are significantly correlated with cancer cell proliferation and migration ability, indicating that *MZF1* may play an important regulatory role in tumorigenesis and progression.

## 4 Discussion

In recent years, with the rapid advancement of technology, the mechanisms by which the immune system interacts with malignant tumor cells have gradually been elucidated, thereby paving the way for diverse therapeutic strategies in cancer treatment. These approaches include chemotherapy, radiotherapy, surgery, immunotherapy, targeted therapy, and endocrine therapy, among others (47). Immunotherapy aims to harness the power of the immune system to overcome these immune evasion strategies, specifically targeting cancer cells. ICB therapy utilizes ICIs to interrupt the binding between checkpoint molecules and their ligands, thereby reactivating suppressed immune cells and restoring the immune system’s ability to target and kill tumor cells (48). In order to accurately predict patient responses to immunotherapy and ensure personalized treatment regimens, there is an urgent need for the development of novel, precise biomarkers to improve the prognosis of cancer patients. In this study, *MZF1* may serve as a novel pan-cancer prognostic biomarker, offering valuable insights into predicting responses to immunotherapy.

According to recent cancer reports, breast cancer has become the most prevalent malignancy among women, with its incidence continuously rising. Statistics indicate that over 1.2 million new cases of breast cancer are diagnosed annually worldwide, with nearly 400,000 deaths attributed to the disease (49). Against this backdrop, the *MZF1* gene, the focus of this study, has been shown to play a pivotal role in various biological processes associated with tumor invasion, metastasis, proliferation, and drug resistance. Its aberrant expression is closely linked to the onset, progression, and prognosis of multiple types of cancer (14). Overexpression of *MZF1* significantly inhibits the proliferation, migration, and clonogenic ability of T-cell lymphomas (50). In prostate cancer, the inhibition of MZF1 expression markedly accelerates tumor cell proliferation (51). In contrast, in colorectal cancer, the overexpression of MZF1 enhances the invasiveness and migratory potential of colon cancer cells (52). In cervical cancer SiHa cells, overexpression of *MZF1* significantly suppresses invasion and migration, whereas in HeLa cells, the effects are entirely reversed (52, 53). A similar dual role of *MZF1* has been observed in gastric cancer, where it functions both as an oncogene and a tumor suppressor (54, 55).

Previous studies have revealed that the role of *MZF1* in tumorigenesis is far from uniform across different cancer types. It can function as a tumor-suppressing gene, yet under certain conditions, it may also act to promote cancer progression. For instance, *MZF1*-mediated regulation of miR-328-3p acts as a tumor suppressor by modulating CD44 expression in the progression of STAD (56). *MZF1* also functions as a tumor suppressor in the hematopoietic compartment (57). In cells overexpressing *MZF1*, significant increases in promoter activity, p55PIK expression, and cell proliferation rates have been observed (58). Both Sp1/Sp3 and *MZF1* are critical transcription factors that regulate N-cadherin promoter activity and expression in osteoblasts (59). In the present study, we highlight the pivotal role of *MZF1* in regulating immune responses, particularly in immune cells such as CD4+ T cells and monocytes/macrophages, suggesting its involvement in the tumor immune microenvironment. These findings provide compelling experimental evidence supporting the investigation of *MZF1*’s role in cancer progression, particularly its contribution to tumor immune regulation and immune cell infiltration. Extensive research has highlighted *MZF1*’s potential as a novel biomarker, suggesting that it could not only aid in the early diagnosis of cancer but also offer new insights into cancer treatment strategies. Future investigations into *MZF1* may provide deeper insights into its complex role in cancer pathogenesis and potentially pave the way for the development of novel therapeutic strategies, particularly in addressing issues related to drug resistance. Although existing studies have demonstrated that *MZF1* binds to the activation site of cyclin-dependent kinase 4 (CDK4) and accelerates the ubiquitination of PD-L1, thereby promoting tumor progression in hepatocellular carcinoma and enhancing resistance to anti-PD-L1 antibodies, its precise mechanisms of action across various cancers remain to be further elucidated (60).

Subsequently, a correlation analysis was conducted between *MZF1* expression and four key survival metrics — OS, PFS, DSS, and mortality. It was observed that *MZF1* exhibited a relatively weak prognostic association with a limited number of cancers. The differential roles of *MZF1* across various cancers may be attributed to the unique biological characteristics of these malignancies, their microenvironments, and the varying functions of *MZF1* in different tumor types. Overall, *MZF1* exhibits significant prognostic value in the majority of cancer patients, emerging as a promising prognostic biomarker, particularly in immune-related cancers. In these cancers, *MZF1* expression may influence the immune microenvironment and tumor immune evasion mechanisms. Thus, evaluating *MZF1* expression aids in the accurate prediction of patient survival and provides valuable support for the development of personalized treatment strategies. While its role in certain cancer types remains unclear, a deeper understanding of the underlying mechanisms of *MZF1* may unveil additional biological features, thereby advancing its clinical application as a prognostic marker.

In subsequent analyses, *MZF1* was found to exhibit the most prevalent genetic alterations in UCS, suggesting that *MZF1* may play a pivotal role in the initiation and progression of this particular tumor type. Further genomic analysis revealed that mutations, CNVs, and changes in *MZF1* expression levels could influence tumor behavior and patient prognosis. In the majority of cancer types, *MZF1* showed a significant positive correlation with tumor malignancy, indicating that higher *MZF1* copy numbers are associated with increased tumor invasiveness, proliferative capacity, and metastatic potential. However, in certain cancers, such as GBM, KIRC, LGG, UCEC, and KIRP, elevated *MZF1* expression did not significantly correlate with improved overall survival. This may suggest that the role of *MZF1* in these cancer types involves more complex or distinct mechanisms. The methylation status of *MZF1* could profoundly impact its function and cancer prognosis, potentially influencing tumor progression through the regulation of gene expression. Specifically, hypermethylation may suppress *MZF1* expression, thereby reducing tumor malignancy and improving patient survival outcomes. The role of *MZF1* across different cancers demonstrates considerable heterogeneity. In certain tumor types, such as UCS, genomic alterations in *MZF1* are more pronounced and may influence tumor progression by altering its expression or function. Conversely, in other cancer types, particularly KIRC, genomic alterations and methylation status of *MZF1* may be more closely associated with patient survival, underscoring its potential as a prognostic factor. Notably, high methylation of *MZF1* could serve as a biomarker for reduced mortality risk, suggesting a potential protective role for *MZF1* in early cancer prognosis. These data further emphasize the potential of *MZF1* as a cancer prognostic biomarker, providing valuable genomic insights into its role across various tumor types. Future research may delve deeper into the relationship between *MZF1* methylation, copy number variations, and their interactions with the tumor microenvironment and immune evasion mechanisms. This could enhance its application in precision medicine and personalized therapeutic strategies.

In recent years, the field of immunotherapy has made rapid advancements, offering new hope to cancer patients. However, despite the initial positive responses of many patients to immunotherapy, a significant portion ultimately faces substantial challenges. Consequently, the widespread adoption of predictive biomarkers, such as TMB, to assess the potential efficacy of immunotherapy in diverse cancer populations has garnered increasing attention (54, 55). Diagnostic analyses related to TMB provide compelling evidence for investigating *MZF1* as a predictive biomarker for immunotherapy response. Moreover, elevated *MZF1* expression levels have been closely associated with prolonged survival, highlighting *MZF1*’s potential as a key biomarker of sensitivity to immune checkpoint blockade therapies. This, in turn, enhances the prospect of more personalized and effective cancer treatments. By integrating *MZF1* with other immune-related biomarkers such as TMB and MSI, it may help identify patients most likely to benefit from immune checkpoint inhibitors, providing invaluable insights for the development of tailored treatment strategies. As the relationship between *MZF1* and immunotherapy efficacy becomes increasingly evident, its potential as a cornerstone in future cancer immunotherapy research grows as well. Future studies should further explore the interactions between *MZF1* and immune evasion mechanisms, as well as its impact within the tumor microenvironment. Such research could pave the way for novel strategies to modulate *MZF1* expression, thereby enhancing the effectiveness of immunotherapies and offering cancer patients more precise and effective therapeutic options. Undoubtedly, this research holds the potential to push the boundaries of cancer immunotherapy, heralding a new chapter in the battle against malignancies.

Through comprehensive bioinformatics enrichment analyses using GSEA and GSVA, it was revealed that *MZF1* may regulate various facets of immune responses and immune activation, influencing the delicate balance between tumor immune evasion and anti-tumor immunity. Notably, in BRCA, *MZF1* expression was found to be associated with the modulation of immune cell functions, suggesting that *MZF1* not only serves as a potential biomarker for tumor growth but also affects the tumor’s response to immunotherapy. These findings lay the foundation for new insights into the role of *MZF1*, indicating that it may indirectly influence tumor initiation, progression, and response to immunotherapy by modulating immune responses and immune cell infiltration within the tumor microenvironment. Thus, future research should delve deeper into the specific mechanisms by which *MZF1* exerts its effects within the tumor immune microenvironment, and further explore its potential to enhance the outcomes of cancer immunotherapy. Such investigations could illuminate new therapeutic strategies aimed at harnessing *MZF1*’s regulatory capabilities to optimize cancer treatment and improve patient prognosis.

Based on drug sensitivity analyses from multiple databases (CellMiner, CTRP, and GDSC), along with further experimental exploration, gain a deeper understanding of the relationship between *MZF1* and various therapeutic agents, as well as its potential in cancer treatment. Notably, *MZF1* demonstrates heightened sensitivity to the following agents: JNK Inhibitor VIII, Elesclomol, Docetaxel, Bleomycin, AZD6482, 17-AAG. These findings suggest that the therapeutic efficacy of these drugs may be modulated by the expression levels of *MZF1*, implying that *MZF1* plays a critical role in mediating the drug’s inhibitory effects on tumor cells. The upregulation or downregulation of *MZF1* may influence the efficacy of these drugs, potentially serving as a predictive biomarker for the therapeutic response to certain anticancer agents. To further validate the interactions between *MZF1* and these anticancer drugs, molecular docking experiments using the Autodock4 tool can be employed. This approach allows for precise measurement of the binding affinity between *MZF1* protein and these pharmacological agents, providing insight into whether *MZF1* might enhance or attenuate the drug’s efficacy through direct molecular interactions. For instance, panobinostat, an HDAC inhibitor, has demonstrated substantial antitumor activity in previous studies and has shown synergistic effects when combined with other drugs. Specifically, co-treatment with panobinostat and parbivastat or tanespimycin has yielded stronger antitumor outcomes, including the inhibition of tumor stem cell activity, malignant proliferation, and metastasis. Such combination therapies may significantly enhance drug effectiveness and provide novel avenues for clinical treatment strategies. Building on the aforementioned findings, it can be hypothesized that panobinostat may potentiate its antitumor effects by modulating the expression or function of *MZF1* (61). Parbivastat, through the inhibition of ARL4C, has been shown to significantly reduce proliferation, invasion, and migration in renal cancer cells (62). Similarly, tanespimycin induces apoptosis, suppressing both tumor cell proliferation and metastasis (63). Therefore, panobinostat, by suppressing *MZF1* expression, may enhance the tumor-suppressive action of these drugs, thereby improving the overall therapeutic response in cancer treatment. This provides new insights and potential strategies for advancing cancer therapies.

While these findings underscore the promising potential of *MZF1* as a prognostic biomarker and its possible role in immunotherapy response, several limitations must be acknowledged. Firstly, this study predominantly relies on bioinformatics analyses, which, while robust, require validation through experimental and clinical research. At present, functional validation predominantly relies on *in vitro* experiments, with confirmation constrained to a singular breast cancer and gastric cancer cell line. There is a notable absence of validation across diverse cancer types, as well as within additional breast and gastric cancer cell lines, animal models, and human cancer biopsies derived from clinical trials. Furthermore, the screening of targeted therapeutics remains at the stage of prediction and preliminary validation, without the implementation of systematic pharmacodynamic evaluations. The potential of *MZF1* as a therapeutic target in cancer treatment warrants further investigation, particularly in cancers where it plays a pivotal role in tumor progression and immune regulation. Expanding these studies could provide crucial insights into *MZF1*’s utility in precision oncology and enhance therapeutic strategies.

For future directions in this research, a deeper investigation into the molecular mechanisms of *MZF1* could be pursued, utilizing technologies such as ChIP-seq and RNA-seq to comprehensively identify its direct target genes and regulatory networks. Additionally, multicenter clinical sample validations should be conducted to assess the clinical utility of *MZF1* as a diagnostic biomarker and prognostic indicator. The development of specific inhibitors and the exploration of combination therapies with immune checkpoint inhibitors could offer new therapeutic avenues. Furthermore, establishing tumor models that more closely mimic human physiological conditions, supported by advanced technologies, could pave the way for personalized treatment prediction systems based on *MZF1* expression profiles, potentially incorporating artificial intelligence algorithms. Collaborative efforts across multiple disciplines, including bioinformatics, chemical biology, and clinical medicine, should be mobilized to further investigate and analyze *MZF1*, accelerating the clinical translation of its research findings. Additionally, exploring the role of *MZF1* in novel areas such as tumor metabolic reprogramming and therapy resistance could open new frontiers for understanding its functions. This study provides systematic evidence to deepen the understanding of *MZF1*’s role in tumor initiation and progression, and through interdisciplinary integration and technological innovation, it holds the potential to transition *MZF1* from a foundational research discovery to a clinical target, offering new strategies and approaches for precision cancer therapy.

In summary, this study provides a comprehensive analysis of the potential role of *MZF1* across various cancers, underscoring its significant promise as a prognostic biomarker, particularly in breast cancer. Through an in-depth examination of experimental data related to immune cell infiltration, tumor microenvironment, TMB and MSI, a clearer understanding of *MZF1*’s involvement in cancer immunotherapy has been achieved. The findings suggest that *MZF1* not only serves as a prognostic predictor for cancer patients but also offers valuable insight into their potential response to immunotherapy, providing a theoretical foundation for the development of personalized treatment strategies. In particular, in the context of breast cancer, the value of *MZF1* as a potential molecular marker has been preliminarily validated. As research advances, the potential of *MZF1* to precisely activate antitumor immune responses and enhance the efficacy of immunotherapies will become increasingly apparent. Thus, as the understanding of *MZF1* deepens, it is poised to play a pivotal role in the future of cancer immunotherapy, facilitating personalized and more effective treatment approaches. This would, in turn, advance the field of cancer immunotherapy and offer renewed hope for a broader range of cancer patients.

## Data Availability

The datasets presented in this article are not readily available. Requests to access the datasets should be directed to jiangchao1212@126.com.
