# Correction: *Myeloid Zinc Finger 1*: insights into its oncogenic potential, prognostic value, and impact on immune microenvironment across cancers

**DOI:** 10.3389/fimmu.2025.1659836

**Published:** 2025-07-25

**Authors:** Wenze Tian, Chun Yu, Xin Xu, Jiaqi Li, Shuangfu Peng, Aijun Sun, Shasha Zhang, Chao Jiang

**Affiliations:** ^1^ Department of Thoracic Surgery, The Affiliated Huaian No.1 People’s Hospital of Nanjing Medical University, Huai’an, China; ^2^ General Surgery Department, Lianshui People’s Hospital Affiliated to Kangda College of Nanjing Medical University, Huai’an, Jiangsu, China; ^3^ Department of Thyroid and Breast Oncological Surgery, Huai’an Second People’s Hospital, The Affiliated Huaian Hospital of Xuzhou Medical University, Huai’an, Jiangsu, China; ^4^ Faculty of Life Science and Food Engineering, Huaiyin Institute of Technology, Huai’an, Jiangsu, China; ^5^ Key Laboratory of Systems Biomedicine, Shanghai Center for Systems Biomedicine, Engineering Research Center of Techniques and Instruments for Diagnosis and Treatment of Congenital Heart Disease, Institute of Developmental and Regenerative Medicine, Xin Hua Hospital, School of Medicine, Shanghai Jiao Tong University, Shanghai, China; ^6^ Department of Oncology, The Affiliated Huaian No.1 People’s Hospital of Nanjing Medical University, Huai’an, China

**Keywords:** *Myeloid Zinc Finger 1*, pan-cancer, tumor immunity, prognostic biomarker, immunotherapy drug susceptibility

## Author affiliation

In the article, there was an error in the affiliations. “Affiliation 3, Department of Thyroid and Breast Oncological Surgery, Huai’an Second People’s Hospital, The Affiliated Huaian Hospital of Xuzhou Medical University, Huai’an, Jiangsu, China”, was erroneously omitted for author “Aijun Sun”. This affiliation has now been added.

Additionally, author “Aijun Sun” was erroneously assigned to affiliation “2, General Surgery Department, Lianshui People’s Hospital Affiliated to Kangda College of Nanjing Medical University, Huai’an, Jiangsu, China”. This affiliation has now been removed.

The original version of this article has been updated.

