# Supplementary material for: Myeloid Zinc Finger 1: insights into its oncogenic potential, prognostic value, and impact on immune microenvironment across cancers
Source: Front Immunol. 2025 Jun 27;16:1591912. doi: 10.3389/fimmu.2025.1591912 (PMC12245674; doi:10.3389/fimmu.2025.1591912)

**Supplementary files**

**Association Between Obesity Indexes and Chronic Kidney Disease Risk: A Double-cohort Prospective Study in the Binhai and UK Biobank**

I Introduction of the Tianjin Chronic Kidney Disease Study

II Supplementary Tables

Supplementary Table 1

Supplementary Table 2

Supplementary Table 3

Supplementary Table 4

Supplementary Table 5

Supplementary Table 6

Supplementary Table 7

Supplementary Table 8

Supplementary Table 9

Supplementary Table 10

Supplementary Table 11

III Supplementary Figures

Supplementary Figure 1

Supplementary Figure 2

Supplementary Figure 3

Supplementary Figure 4

Supplementary Figure 5

**Introduction of the Tianjin Chronic Kidney Disease Study**

**Cohort name and international clinical trial registration:** Tianjin Chronic Disease Cohort Study; registration number, ChiCTR1900023701.

**Project leader (lead institution):** Pei Yu (Chu Hsien-I Memorial Hospital & Tianjin Institute of Endocrinology, Tianjin Medical University)

**Project description:** The study is an open cohort study that started in January 2013. It aims to explore the intrinsic patterns in the occurrence and progression of chronic diseases in the elderly population, identify key risk factors, and provide a scientific basis for effective prevention and control of the spread of chronic diseases.

**Main objectives:** Prediction, prevention and control strategy development of chronic diseases in the elderly

**Study design:** Open prospective cohort study in adult population.

**Inclusion criteria:** Adults aged 18 years or older.

**Exclusion criteria:** those who were disabled, semi-disabled, refused or unable to participate in regular medical check-up surveys.

**Population characteristics:** Cohort of adults aged 18 years or older from different levels of physical examination centers and communities in Binhai New Area, Tianjin. The population composition was matched for age, gender and occupation.

**Completed participants per year/total number of participants:** more than 300,000/2 million.

**Starting and ending years and follow-up intervals:** 2013 to present; at least 1 follow-up visit every 1 year.

**Medical examination and measurement indicators:** age, gender, smoking, alcohol consumption, occupation, education, blood pressure, heart rate, height, weight, waist circumference, ECG, abdominal ultrasound, blood routine (hemoglobin, platelets, lymphocytes, etc.), urine routine, liver and kidney function, electrolytes, fasting blood glucose, 2h postprandial blood glucose, triglycerides, total cholesterol, HDL, LDL, blood uric acid, albumin, glutamate transaminase, glutamic acid transaminase, etc.; exercise frequency, time and mode, dietary habits, etc.

**Medication indicators:** types of medications for various chronic diseases, medication dosage and frequency, compliance.

**Outcome indicators:** diabetes, hypertension, psychosis, hyperlipidemia, fatty liver, atrial fibrillation, disability and semi-disability, cardiovascular and cerebrovascular diseases, cancer, healthy life expectancy and all-cause mortality, etc.

**Data collection:** Experienced professional physicians and nurses from community and medical examination centers will complete data collection and fill in the system of Tianjin Community Health Service Center.

**Data management:** Uniformly uploaded to system of Tianjin Community Health Service Center for management, and data can be downloaded by logging into this system.

**Supplementary Tables**

**Supplementary Table 1.** The calculation formulas of obesity indexes.

| **Obesity indexes** | **Gender** | **Calculation formula** |
| --- | --- | --- |
| CVAI | Male | -267.93 + 0.68 × age (years) + 0.03 × BMI (kg/m^2^) + 4.00 × WC (cm) + 22.00 × Log10 (TG (mmol/L)) – 16.32 × HDL-c (mmol/L). |
|  | Female | -187.32 + 1.71 × age (years) + 4.23 × BMI (kg/m^2^) 1.12 × WC (cm) + 39.76 × Log10 (TG (mmol/L)) – 11.66 × HDL-c (mmol/L). |
| BAI |  | [Hips (cm) / height (m)^1.5] -18 |
| BMI |  | weight (kg) / height (m) ^2 |
| WHtR |  | WC (cm) / height (cm) |
| ABSI |  | WC (m) /(BMI ^2/3 (kg/m^2^) ×height ^1/2 (m)) |
| BRI |  | 364.2 – 365.5 × [1 – (WC (m) /2π) ^2/(0.5 × height (m)) ^2] ^1/2 |
| LAP | Male | (WC (cm) − 65)×TG (mmol/L) |
|  | Female | (WC (cm) − 58) × TG (mmol/L) |
| VAI | Male | WC (cm) / (39.68 + (1.88 × BMI (kg/m^2^)) × (TG (mmol/L) / 1.03) × (1.31 / HDL-c (mmol/L)) |
|  | Female | WC (cm) / (36.58 + (1.89 × BMI (kg/m^2^)) × (TG (mmol/L) / 0.81) × (1.52 / HDL-c (mmol/L)) |

**Supplementary Table 2.** Baseline characteristics of participants stratified by gender (Binhai cohort).

| Characteristics | Men | Women | *p* value |
| --- | --- | --- | --- |
|  | (n = 57903) | (n = 68206) |  |
| Age, years | 68.95 (6.70) | 68.32 (6.31) | <0.001^a^ |
| BMI, kg/m^2^ | 24.89 (2.89) | 24.85 (3.21) | 0.013^a^ |
| WC, cm | 87.83 (8.00) | 84.09 (8.18) | <0.001^a^ |
| WHtR | 0.51 (0.05) | 0.53 (0.05) | <0.001^a^ |
| CVAI | 111.52 (34.75) | *118.69 (27.29)* | <0.001^a^ |
| VAI | 1.53 (0.98) | 2.32 (1.40) | <0.001^a^ |
| ABSI | 0.0790 (0.0049) | 0.0786 (0.0052) | <0.001^a^ |
| LAP | 34.62 (23.17) | 43.53 (26.29) | <0.001^a^ |
| BRI | 3.68 (0.95) | 3.99 (1.11) | <0.001^a^ |
| SBP, mmHg | 129.06 (12.39) | 128.20 (12.78) | <0.001^a^ |
| DBP, mmHg | 79.36 (7.73) | 78.06 (8.13) | <0.001^a^ |
| Laboratory tests |  |  |  |
| FBG, mmol/L | 5.67 (0.99) | 5.64 (0.98) | <0.001^a^ |
| HGB, g/L | 147.92 (13.28) | 135.26 (10.69) | <0.001^a^ |
| TC, mmol/L | 4.94 (0.95) | 5.42 (1.03) | <0.001^a^ |
| TG, mmol/L | 1.47 (0.71) | 1.63 (0.74) | <0.001^a^ |
| HDL-C, mmol/L | 1.36 (0.35) | 1.45 (0.36) | <0.001^a^ |
| LDL-C, mmol/L | 2.70 (0.87) | 2.98 (0.94) | <0.001^a^ |
| TBIL, μmol/L | 14.82 (5.28) | 13.18 (4.71) | <0.001^a^ |
| ALT, U/L | 22.51 (16.92) | 21.36 (18.95) | <0.001^a^ |
| AST, U/L | 22.47 (11.51) | 22.3 (11.14) | 0.009^a^ |
| Cr, mol/L | 77.51 (13.84) | 62 (11.43) | <0.001^a^ |
| eGFR, ml/min/1.73m² | 89.26 (15.18) | 84.35 (12.19) | <0.001^a^ |
| BUN, mg/dL | 5.65 (1.36) | 5.29 (1.28) | <0.001^a^ |
| Smoking |  |  |  |
| Current | 19562 (33.78) | 6519 (9.56) | <0.001^b^ |
| Never | 30560 (52.78) | 60100 (88.11) |  |
| Quit | 7782 (13.44) | 1588 (2.33) |  |
| Drinking |  |  |  |
| Never | 31534 (54.46) | 66418 (97.38) | <0.001^b^ |
| 1/week | 13232 (22.85) | 1326 (1.94) |  |
| 1-7/week | 3862 (6.67) | 135 (0.20) |  |
| >7/week | 9276 (16.02) | 328 (0.48) |  |
| Exercise |  |  |  |
| Never | 12798 (22.10) | 17571 (25.76) | <0.001^b^ |
| 1/week | 3211 (5.55) | 4175 (6.12) |  |
| 1-7/week | 9825 (16.97) | 11532 (16.91) |  |
| >7/week | 32070 (55.38) | 34929 (51.21) |  |
| Dietary conditions |  |  |  |
| Balanced diet | 55228 (95.38) | 64035 (93.88) | <0.001^b^ |
| Imalanced diet | 2676 (4.62) | 4172 (6.12) |  |

**Supplementary Table 3.** Baseline characteristics of participants stratified by outcome (Binhai cohort).

| Characteristics | Incident chronic kidney disease | | *p* value |
| --- | --- | --- | --- |
|  | No (n = 111674) | Yes (n = 14435) |  |
| Age, years | 68.27 (6.35) | 71.22 (7.05) | <0.001^a^ |
| BMI, kg/m^2^ | 24.83 (3.05) | 25.18 (3.17) | <0.001^a^ |
| WC, cm | 85.71 (8.26) | 86.58 (8.64) | <0.001^a^ |
| WHtR | 0.52 (0.05) | 0.53 (0.05) | <0.001^a^ |
| CVAI | 114.42 (30.99) | 123.00 (31.36) | <0.001^a^ |
| VAI | 1.95 (1.28) | 2.06 (1.32) | <0.001^a^ |
| ABSI | 0.0787 (0.0050) | 0.0791 (0.0051) | <0.001^a^ |
| LAP | 39.03 (25.09) | 42.63 (26.64) | <0.001^a^ |
| BRI | 3.83 (1.04) | 4.03 (1.12) | <0.001^a^ |
| SBP, mmHg | 128.34 (12.54) | 130.54 (12.90) | <0.001^a^ |
| DBP, mmHg | 78.61 (7.78) | 79.02 (9.31) | <0.001^a^ |
| Laboratory tests |  |  |  |
| FBG, mmol/L | 5.63 (0.97) | 5.79 (1.08) | <0.001^a^ |
| HGB, g/L | 141.29 (13.44) | 139.36 (13.89) | <0.001^a^ |
| TC, mmol/L | 5.20 (1.02) | 5.20 (1.04) | 0.831^a^ |
| TG, mmol/L | 1.55 (0.73) | 1.61 (0.74) | <0.001^a^ |
| HDL-C, mmol/L | 1.41 (0.36) | 1.41 (0.37) | 0.192^a^ |
| LDL-C, mmol/L | 2.85 (0.92) | 2.81 (0.95) | <0.001^a^ |
| TBIL, μmol/L | 13.94 (5.05) | 13.86 (5.03) | 0.05^a^ |
| ALT, U/L | 21.84 (18.02) | 22.27 (18.37) | 0.006^a^ |
| AST, U/L | 22.33 (11.33) | 22.78 (11.2) | 0.009^a^ |
| Cr,mol/L | 69.06 (14.47) | 73.65 (14.63) | <0.001^a^ |
| eGFR,ml/min/1.73m² | 87.51 (13.65) | 79.56 (13.45) | <0.001^a^ |
| BUN,mg/dL | 5.42 (1.32) | 5.75 (1.41) | <0.001^a^ |
| Smoking |  |  |  |
| Current | 23400 (20.95) | 2681 (18.57) | <0.001^b^ |
| Never | 80047 (71.68) | 10613 (73.52) |  |
| Quit | 8228 (7.37) | 1142 (7.91) |  |
| Drinking |  |  |  |
| Never | 86248 (77.23) | 11704 (81.07) | <0.001^b^ |
| 1/week | 13154 (11.78) | 1404 (9.73) |  |
| 1-7/week | 3631 (3.25) | 366 (2.54) |  |
| >7/week | 8642 (7.74) | 962 (6.66) |  |
| Exercise |  |  |  |
| Never | 27079 (24.25) | 3290 (22.79) | <0.001^b^ |
| 1/week | 6521 (5.84) | 865 (5.99) |  |
| 1-7/week | 18735 (16.78) | 2622 (18.16) |  |
| >7/week | 59340 (53.13) | 7659 (53.06) |  |
| Dietary conditions |  |  |  |
| Balanced diet | 105713 (94.66) | 13550 (93.86) | <0.001^b^ |
| Imalanced diet | 5962 (5.34) | 886 (6.14) |  |

**Supplementary Table 4.** Baseline characteristics stratified by the quartile of CVAI (U.K. Biobank cohort).

| **Characteristics** | **1st** | **2nd** | **3rd** | **4th** | ***p* value** |  |
| --- | --- | --- | --- | --- | --- | --- |
|  | **<85.94 (n = 89723)** | **85.94-118.78 (n = 89727)** | **118.78-153.63 (n = 89746)** | **>153.63 (n = 89722)** |  |  |
| Male | 18822±20.98 | 34138±38.05 | 48295±53.81 | 63309±70.56 | <0.001^a^ |  |
| Age, years | 52.30±7.74 | 56.76±7.82 | 58.03±7.74 | 58.77±7.46 | <0.001^a^ |  |
| Height, cm | 165.91±7.85 | 167.21±8.95 | 169.23±9.47 | 172.02±9.56 | <0.001^a^ |  |
| Weight, kg | 63.14±8.20 | 71.96±8.66 | 80.56±8.97 | 96.21±13.49 | <0.001^a^ |  |
| BMI, kg/m2 | 22.90±2.22 | 25.72±2.27 | 28.17±2.68 | 32.62±4.60 | <0.001^a^ |  |
| WC, cm | 75.33±6.23 | 84.98±5.69 | 93.41±5.16 | 106.96±8.78 | <0.001^a^ |  |
| WHtR | 0.45±0.04 | 0.51±0.03 | 0.55±0.03 | 0.62±0.06 | <0.001^a^ |  |
| VAI | 1.07±0.59 | 1.73±1.03 | 2.43±1.54 | 3.27±2.11 | <0.001^a^ |  |
| ABSI | 0.0727±0.0046 | 0.0756±0.0047 | 0.0779±0.0046 | 0.0803±0.0047 | <0.001^a^ |  |
| LAP | 16.97±9.24 | 36.56±16.89 | 60.78±28.85 | 103.78±56.24 | <0.001^a^ |  |
| BRI | 2.57±0.61 | 3.55±0.63 | 4.43±0.73 | 6.03±1.42 | <0.001^a^ |  |
| Hips, cm | 95.98±5.65 | 100.63±5.63 | 104.57±6.22 | 112.03±9.54 | <0.001^a^ |  |
| WHTR | 0.79±0.06 | 0.85±0.06 | 0.90±0.06 | 0.96±0.07 | <0.001^a^ |  |
| BAI | 27.05±3.56 | 28.77±4.31 | 29.80±5.17 | 32.04±6.84 | <0.001^a^ |  |
| SBP, mmHg | 130.96±18.88 | 139.15±19.19 | 142.99±18.81 | 145.62±18.44 | <0.001^a^ |  |
| DBP, mmHg | 77.69±10.14 | 81.35±10.17 | 83.83±10.18 | 86.00±10.36 | <0.001^a^ |  |
| Laboratory tests |  |  |  |  |  |  |
| FBG, mmol/L | 5.43 (0.86) | 5.58 (0.94) | 5.70 (0.99) | 5.90 (1.08) | <0.001^a^ |  |
| ALB, g/L | 140.59 (13.69) | 140.47 (13.45) | 140.76 (13.32) | 142.46 (13.49) | <0.001^a^ |  |
| TP, g/L | 72.42±4.17 | 72.49±4.11 | 72.62±4.06 | 72.57±4.02 | <0.001^a^ |  |
| TC, mmol/L | 5.62±1.02 | 5.86±1.11 | 5.83±1.17 | 5.53±1.21 | <0.001^a^ |  |
| TG, mmol/L | 1.07±0.45 | 1.54±0.71 | 1.96±0.97 | 2.38±1.22 | <0.001^a^ |  |
| HDL-C, mmol/L | 1.73±0.39 | 1.52±0.34 | 1.36±0.30 | 1.20±0.27 | <0.001^a^ |  |
| LDL-C, mmol/L | 3.37±0.76 | 3.67±0.85 | 3.71±0.89 | 3.52±0.91 | <0.001^a^ |  |
| TBIL, μmol/L | 9.27±4.59 | 8.97±4.34 | 9.09±4.32 | 9.17±4.24 | <0.001^a^ |  |
| ALT, U/L | 17.84±9.92 | 21.29±12.27 | 24.97±13.96 | 29.74±16.55 | <0.001^a^ |  |
| AST, U/L | 24.19±9.16 | 25.43±9.89 | 26.56±9.72 | 28.50±11.21 | <0.001^a^ |  |
| Cr, mol/L | 66.16±11.99 | 70.16±13.69 | 73.76±14.53 | 77.11±15.57 | <0.001^a^ |  |
| eGFR, ml/min/1.73m² | 96.27±12.93 | 92.66±14.00 | 91.31±14.84 | 90.91±16.18 | <0.001^a^ |  |
| BUN, mg/dL | 5.01±1.22 | 5.34±1.25 | 5.49±1.28 | 5.64±1.39 | <0.001^a^ |  |
| UA, umol/L | 253.97±60.71 | 291.61±67.74 | 325.26±71.26 | 361.66±75.03 | <0.001^a^ |  |
| Smoking, n (%) |  |  |  |  |  |  |
| Current | 9381 (10.46) | 9163 (10.21) | 9301 (10.36) | 9703 (10.81) | <0.001^b^ |  |
| Never | 55419 (61.77) | 51952 (57.90) | 47948 (53.43) | 41187 (45.91) |  |  |
| Quit | 24923 (27.78) | 28612 (31.89) | 32497 (36.21) | 38832 (43.28) |  |  |
| Drinking, n (%) |  |  |  |  |  |  |
| Current | 83700 (93.29) | 83295 (92.83) | 82650 (92.09) | 81649 (91.00) | <0.001^b^ |  |
| Never | 3405 (3.80) | 3747 (4.18) | 3993 (4.45) | 4015 (4.47) |  |  |
| Quit | 2618 (2.92) | 2685 (2.99) | 3103 (3.46) | 4058 (4.52) |  |  |
| CHD, n (%) |  |  |  |  |  |  |
| Yes | 8184 (25.96) | 7517 (23.83) | 7101 (22.53) | 7566 (24.00) | <0.001^b^ |  |
| No | 1795 (5.69) | 1965 (6.23) | 1817 (5.76) | 1809 (5.74) |  |  |
| Hypertension, n (%) |  |  |  |  |  |  |
| Yes | 9601 (10.70) | 18573 (20.70) | 26394 (29.41) | 39321 (43.83) | <0.001^b^ |  |
| No | 80122 (89.30) | 71154 (79.30) | 63352 (70.59) | 50401 (56.17) |  |  |
| Stroke, n (%) |  |  |  |  |  |  |
| Yes | 548 (0.61) | 954 (1.06) | 1406 (1.57) | 2078 (2.32) | <0.001^b^ |  |
| No | 89175 (99.39) | 88773 (98.94) | 88340 (98.43) | 87644 (97.68) |  |  |
| Diabetes, n (%) |  |  |  |  |  |  |
| Yes | 1094 (1.22) | 1968 (2.19) | 3809 (4.24) | 10587 (11.80) | <0.001^b^ |  |
| No | 88629 (98.78) | 87759 (97.81) | 85937 (95.76) | 79135 (88.20) |  |  |
| Cancer, n (%) |  |  |  |  |  |  |
| Yes | 5861 (6.53) | 6928 (7.72) | 7130 (7.94) | 6938 (7.73) | <0.001^b^ |  |
| No | 83862 (93.47) | 82799 (92.28) | 82616 (92.06) | 82784 (92.27) |  |  |
| new cases of CKD, n (%) | 2410 (2.69) | 4445 (4.95) | 6177 (6.88) | 9821 (10.95) | <0.001^c^ |  |

^a^Analysis of Variance, ^b^Chi-square test. Normally distributed data are expressed as mean and SDs, non-normally distributed data are expressed as median and quartiles, the rest are expressed as counts and percentages. SBP: systolic blood pressure. DBP: diastolic blood pressure. FBG: fasting blood glucose. ALB: albumin. TP: total protein. TC: total cholesterol. TG: triglyceride. HDL-c: high-density lipoprotein cholesterol. LDL-c: low-density lipoprotein cholesterol. TBIL: total bilirubin. AST: aspartate transaminase. ALT: alanine transaminase. Cr: creatinine. eGFR: estimated glomerular filtration rate. BUN: blood urea nitrogen.UA: uric acid. CHD: coronary heart disease.

**Supplementary Table 5.** Baseline characteristics of participants stratified by gender (U.K. Biobank cohort).

| Characteristics | Man | Women | *p value* |
| --- | --- | --- | --- |
|  | (n = 164564) | (n = 194354) |  |
| Age, years | 56.64±8.20 | 56.32±7.99 | <0.001^a^ |
| Height, cm | 175.74±6.82 | 162.54±6.29 | <0.001^a^ |
| Weight, kg | 85.86±14.24 | 71.29±13.89 | <0.001^a^ |
| CVAI | 142.81±49.66 | 104.59±44.15 | <0.001^a^ |
| BMI, kg/m2 | 27.77±4.19 | 26.99±5.11 | <0.001^a^ |
| WC, cm | 96.81±11.22 | 84.55±12.42 | <0.001^a^ |
| WHtR | 0.55±0.06 | 0.52±0.08 | <0.001^a^ |
| VAI | 2.30±1.74 | 1.98±1.55 | <0.001^a^ |
| ABSI | 0.0798±0.0041 | 0.0739±0.0050 | <0.001^a^ |
| LAP | 65.91±50.67 | 44.88±39.71 | <0.001^a^ |
| BRI | 4.45±1.41 | 3.88±1.64 | <0.001^a^ |
| Hips, cm | 103.38±7.53 | 103.24±10.26 | <0.001^a^ |
| WHTR | 0.93±0.06 | 0.82±0.07 | <0.001^a^ |
| BAI | 26.45±3.55 | 31.92±5.47 | <0.001^a^ |
| SBP, mmHg | 142.70±18.47 | 137.12±20.21 | <0.001^a^ |
| DBP, mmHg | 84.05±10.54 | 80.67±10.53 | <0.001^a^ |
| Laboratory tests |  |  |  |
| FBG, mmol/L | 5.17±1.35 | 5.06±1.03 | <0.001^a^ |
| ALB, g/L | 45.56±2.60 | 44.98±2.59 | <0.001^a^ |
| TP, g/L | 72.64±4.08 | 72.43±4.10 | <0.001^a^ |
| TC, mmol/L | 5.50±1.12 | 5.88±1.12 | <0.001^a^ |
| TG, mmol/L | 1.96±1.13 | 1.54±0.85 | <0.001^a^ |
| HDL-C, mmol/L | 1.28±0.31 | 1.60±0.38 | <0.001^a^ |
| LDL-C, mmol/L | 3.49±0.86 | 3.63±0.87 | <0.001^a^ |
| TBIL, μmol/L | 10.29±4.83 | 8.14±3.67 | <0.001^a^ |
| ALT, U/L | 27.40±15.17 | 20.13±12.18 | <0.001^a^ |
| AST, U/L | 28.20±10.88 | 24.45±9.14 | <0.001^a^ |
| Cr, mol/L | 81.07±13.11 | 63.95±10.63 | <0.001^a^ |
| eGFR, ml/min/1.73m² | 94.41±16.37 | 91.41±12.93 | <0.001^a^ |
| BUN, mg/dL | 5.57±1.32 | 5.20±1.27 | <0.001^a^ |
| UA, umol/L | 353.65±70.72 | 269.58±65.02 | <0.001^a^ |
| Smoking, n (%) |  |  |  |
| Current | 20437 (12.42) | 17111 (8.80) | <0.001^b^ |
| Never | 80674 (49.02) | 115832 (59.60) |  |
| Quit | 63453 (38.56) | 61411 (31.60) |  |
| Drinking, n (%) |  |  |  |
| Current | 154715 (94.02) | 176579 (90.85) | <0.001^b^ |
| Never | 4269 (2.59) | 10891 (5.60) |  |
| Quit | 5580 (3.39) | 6884 (3.54) |  |
| Hypertension, n (%) |  |  |  |
| Yes | 10838 (6.59) | 4326 (2.23) | <0.001^b^ |
| No | 153726 (93.41) | 190028 (97.77) |  |
| HBP, n (%) |  |  |  |
| Yes | 48512 (29.48) | 45377 (23.35) | <0.001^b^ |
| No | 116052 (70.52) | 148977 (76.65) |  |
| Stroke, n (%) |  |  |  |
| Yes | 2944 (1.79) | 2042 (1.05) | <0.001^b^ |
| No | 161620 (98.21) | 192312 (98.95) |  |
| Diabetes, n (%) |  |  |  |
| Yes | 10699 (6.50) | 6759 (3.48) | <0.001^b^ |
| No | 153865 (93.50) | 187595 (96.52) |  |
| Cancer, n (%) |  |  |  |
| Yes | 9706 (5.90) | 17151 (8.82) | <0.001^b^ |
| No | 154858 (94.10) | 177203 (91.18) |  |
| new cases of CKD, n (%) |  |  |  |
|  | 12153 (7.38) | 10700 (5.51) | <0.001^c^ |

**Supplementary Table 6.** Baseline characteristics of participants stratified by outcome (U.K. Biobank cohort).

|  | CKD occurred at the end point | | *p value* |  |
| --- | --- | --- | --- | --- |
|  | No (n =336065) | Yes (n = 22853) |  |  |
| Age, years | 56.17±8.08 | 60.78±6.96 | <0.001^a^ |  |
| Height, cm | 168.59±9.28 | 168.61±9.28 | 0.854^a^ |  |
| Weight, kg | 77.64±15.68 | 82.86±16.90 | <0.001^a^ |  |
| CVAI | 120.34±49.84 | 148.26±52.68 | <0.001^a^ |  |
| BMI, kg/m2 | 27.23±4.66 | 29.09±5.26 | <0.001^a^ |  |
| WC, cm | 89.79±13.23 | 95.80±14.04 | <0.001^a^ |  |
| WHtR | 0.53±0.07 | 0.57±0.08 | <0.001^a^ |  |
| VAI | 2.10±1.62 | 2.58±1.94 | <0.001^a^ |  |
| ABSI | 0.0765±0.0055 | 0.0782±0.0054 | <0.001^a^ |  |
| LAP | 53.42±45.44 | 70.71±54.59 | <0.001^a^ |  |
| BRI | 4.09±1.54 | 4.86±1.79 | <0.001^a^ |  |
| Hips, cm | 103.14±9.01 | 105.74±10.12 | <0.001^a^ |  |
| WHTR | 0.87±0.09 | 0.90±0.09 | <0.001^a^ |  |
| BAI | 29.34±5.37 | 30.55±6.08 | <0.001^a^ |  |
| SBP, mmHg | 139.34±19.53 | 144.65±20.41 | <0.001^a^ |  |
| DBP, mmHg | 82.17±10.64 | 83.93±11.02 | <0.001^a^ |  |
| Laboratory tests |  |  |  |  |
| FBG, mmol/L | 5.09±1.13 | 5.47±1.83 | <0.001^a^ |  |
| ALB, g/L | 45.28±2.60 | 44.70±2.71 | <0.001^a^ |  |
| TP, g/L | 72.53±4.07 | 72.47±4.35 | 0.023^a^ |  |
| TC, mmol/L | 5.72±1.13 | 5.45±1.25 | <0.001^a^ |  |
| TG, mmol/L | 1.72±1.00 | 1.96±1.09 | <0.001^a^ |  |
| HDL-C, mmol/L | 1.46±0.38 | 1.35±0.37 | <0.001^a^ |  |
| LDL-C, mmol/L | 3.58±0.86 | 3.41±0.94 | <0.001^a^ |  |
| TBIL, μmol/L | 9.13±4.37 | 9.10±4.40 | 0.330^a^ |  |
| ALT, U/L | 23.37±14.03 | 24.71±15.07 | <0.001^a^ |  |
| AST, U/L | 26.09±10.03 | 27.33±11.76 | <0.001^a^ |  |
| Cr, mol/L | 71.11±13.77 | 81.96±21.11 | <0.001^a^ |  |
| eGFR, ml/min/1.73m² | 93.56±14.09 | 81.37±18.09 | <0.001^a^ |  |
| BUN, mg/dL | 5.32±1.25 | 6.12±1.08 | <0.001^a^ |  |
| UA, umol/L | 305.83±78.53 | 341.88±87.30 | <0.001^a^ |  |
| Smoking, n (%) | 336065 | 22853 |  |  |
| Current | 34912 (10.39) | 2636 (11.53) | <0.001^b^ |  |
| Never | 185698 (55.26) | 10808 (47.29) |  |  |
| Quit | 115455 (34.35) | 9409 (41.17) |  |  |
| Drinking, n (%) |  |  |  |  |
| Current | 311021 (92.55) | 20273 (88.71) | <0.001^b^ |  |
| Never | 13794 (4.10) | 1366 (5.98) |  |  |
| Quit | 11250 (3.35) | 1214 (5.31) |  |  |
| CHD, n (%) |  |  |  |  |
| Yes | 12494 (3.72) | 2670 (11.68) | <0.001^b^ |  |
| No | 323571 (96.28) | 20183 (88.32) |  |  |
| Hypertension, n (%) |  |  |  |  |
| Yes | 83321 (24.79) | 10568 (46.24) | <0.001^b^ |  |
| No | 252744 (75.21) | 12285 (53.76) |  |  |
| Stroke, n (%) |  |  |  |  |
| Yes | 4185 (1.25) | 801 (3.51) | <0.001^b^ |  |
| No | 331880 (98.75) | 22052 (96.49) |  |  |
| Diabetes, n (%) |  |  |  |  |
| Yes | 14168 (4.22) | 3290 (14.40) | <0.001^b^ |  |
| No | 321897 (95.78) | 19563 (85.60) |  |  |
| Cancer, n (%) |  |  |  |  |
| Yes | 24303 (7.23) | 2554 (11.18) | <0.001^b^ |  |
| No | 311762 (92.77) | 20299 (88.82) |  |  |

**Supplementary Table 7.** Hazard Ratio (HR) and 95% confidence intervals (CI) of 3-year incident chronic kidney disease for the VAI in various subgroups.

| Subgroup | N (cases) | HR (95%CI) | *p* | *p* for Interaction |
| --- | --- | --- | --- | --- |
| Gender |  |  |  | ＜0.001 |
| Men | 57903 (5693) | 1.007 (0.939-1.081) | 0.838 |  |
| Women | 68206 (8742) | 0.996 (0.950-1.043) | 0.859 |  |
| Age |  |  |  | 0.253 |
| ＜65 | 36932 (2589) | 0.996 (0.917-1.082) | 0.928 |  |
| 65-70 | 48014 (4785) | 0.962 (0.903-1.025) | 0.236 |  |
| 70-75 | 22340 (3129) | 0.929 (0.856-1.007) | 0.073 |  |
| ＞75 | 18823 (3932) | 0.991 (0.920-1.068) | 0.819 |  |
| Hypertension |  |  |  | 0.218 |
| Yes | 63501 (9181) | 0.964 (0.912-1.009) | 0.112 |  |
| No | 62608 (5254) | 0.995 (0.934-1.061) | 0.889 |  |
| Diabetes |  |  |  | 0.151 |
| Yes | 22571 (3459) | 0.921 (0.854-0.993) | 0.031 |  |
| No | 103538 (10976) | 0.990 (0.948-1.033) | 0.640 |  |
| Stroke |  |  |  | 0.694 |
| Yes | 3343 (518) | 1.031 (0.839-1.265) | 0.773 |  |
| No | 122766 (13917) | 0.969 (0.933-1.007) | 0.105 |  |

**Supplementary Table 8.** Hazard Ratio (HR) and 95% confidence intervals (CI) of 3-year incident chronic kidney disease for the ABSI in various subgroups.

| subgroup | N | HR (95%CI) | *p* | *p* for Interaction |
| --- | --- | --- | --- | --- |
| Gender |  |  |  | 0.357 |
| Men | 57903(5693) | 1.043 (0.998-1.100) | 0.130 |  |
| Women | 68206(8742) | 1.011 (0.971-1.054) | 0.584 |  |
| Age |  |  |  | 0.224 |
| ＜65 | 36932 (2589) | 1.059 (0.976-1-150) | 0.169 |  |
| 65-70 | 48014 (4785) | 1.044 (0.983-1.108) | 0.159 |  |
| 70-75 | 22340 (3129) | 1.012 (0.945-1.084) | 0.723 |  |
| ＞75 | 18823 (3932) | 1.005 (0.949-1.065) | 0.858 |  |
| Hypertension |  |  |  | 0.921 |
| Yes | 63501 (9181) | 1.032 (0.991-1.076) | 0.128 |  |
| No | 62608 (5254) | 1.011 (0.959-1.066) | 0.685 |  |
| Diabetes |  |  |  | 0.986 |
| Yes | 22571 (3459) | 1.072 (1.003-1.145) | 0.039 |  |
| No | 103538 (10976) | 1.010 (0.973-1.049) | 0.595 |  |
| Stroke |  |  |  | 0.409 |
| Yes | 3343 (518) | 1.164 (0.985-1.374) | 0.075 |  |
| No | 122766 (13917) | 1.022 (0.988-1.056) | 0.203 |  |

**Supplementary Table 9.** The self-reported ethnic origins of U.K. Biobank participants (U.K. Biobank cohort).

| Ethnicity | U.K. Biobank cohort (n = 358,918) | |
| --- | --- | --- |
|  | No. of Persons | % |
| White^a^ | 339,934 | 94.7 |
| Black or black British^b^ | 4,920 | 1.4 |
| Asian | 7,735 | 2.2 |
| Other ethnic group | 6,329 | 1.8 |

Abbreviation: U.K., United Kingdom.

^a^ Included white British, white Irish, and other white background.

^b^ Included Caribbean, African, and other black background.

**Supplementary Table 10.** The stratified analyses of the associations between obesity indexes and risk of chronic kidney disease (CKD) by ethnic subgroups in the U.K. Biobank cohort.

| **Ethnicity** | **N** | **Model 1** | **Model 2** | **Model 3** |
| --- | --- | --- | --- | --- |
|  |  | **HR (95%CI)** | **HR (95%CI)** | **HR (95%CI)** |
| **White^a^** | 339,934 | 2.55 (2.49-2.61) | 2.19 (2.13-2.25) | 1.47 (1.42-1.53) |
| *p* for trend |  | <0.001 | <0.001 | <0.001 |
| **Black or black British^b^** | 4,920 | 2.44 (2-2.98) | 1.89 (1.51-2.37) | 1.3 (0.98-1.71) |
| *p* for trend |  | <0.001 | <0.001 | 0.068 |
| **Asian** | 7,735 | 2.88 (2.45-3.38) | 2.13 (1.76-2.57) | 1.29 (1.02-1.63) |
| *p* for trend |  | <0.001 | <0.001 | 0.031 |
| **Other ethnic group** | 6,329 | 2.64 (2.22-3.14) | 2.28 (1.87-2.79) | 1.71 (1.34-2.18) |
| *p* for trend |  | <0.001 | <0.001 | <0.001 |

Abbreviation: U.K., United Kingdom.

^a^ Included white British, white Irish, and other white background.

^b^ Included Caribbean, African, and other black background.

**Supplementary Table 11.** The risk of CKD per CAVI-every 100 unit at 35 months follow-up in the U.K. Biobank cohort.

| **CAVI** | **Model 1** | **Model 2** | **Model 3** |
| --- | --- | --- | --- |
|  | **HR (95%CI)** | **HR (95%CI)** | **HR (95%CI)** |
|  | 2.69 (2.5-2.89) | 2.27 (2.09-2.47) | 1.48 (1.33-1.65) |
| p for trend | <0.001 | <0.001 | <0.001 |

**Supplementary Figure 1.** Spearman correlation analysis between obesity indexes and renal function (U.K. Biobank cohort).


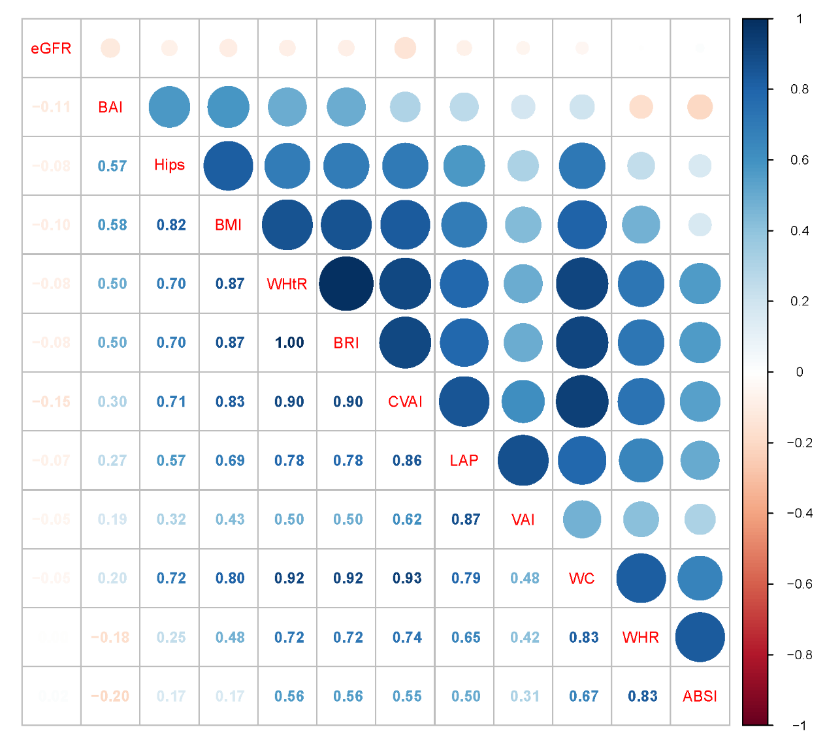


**Supplementary Figure 2.** The dose-response relationships between obesity indexes and risk of CKD (U.K. Biobank cohort). (A) CVAI, Chinese visceral adiposity index. (B) VAI, visceral adiposity index. (C) BMI, body mass index. (D) WHtR, waist-height ratio. (E) ABSI, a body shape index. (F) LAP, lipid accumulation product. (G) WC, waist circumference. (H) BAI, Body adiposity index. (I) BRI, body roundness index. (J) Hips, Hip circumference. (K) WHR, Waist to hip ratio. Adjusted for age, gender, SBP, DBP, LDL-c, HDL-c, TC, TG, Cr at baseline, eGFR at baseline, smoking, drinking, history of diabetes, hypertension, coronary heart disease, cancer and stroke.


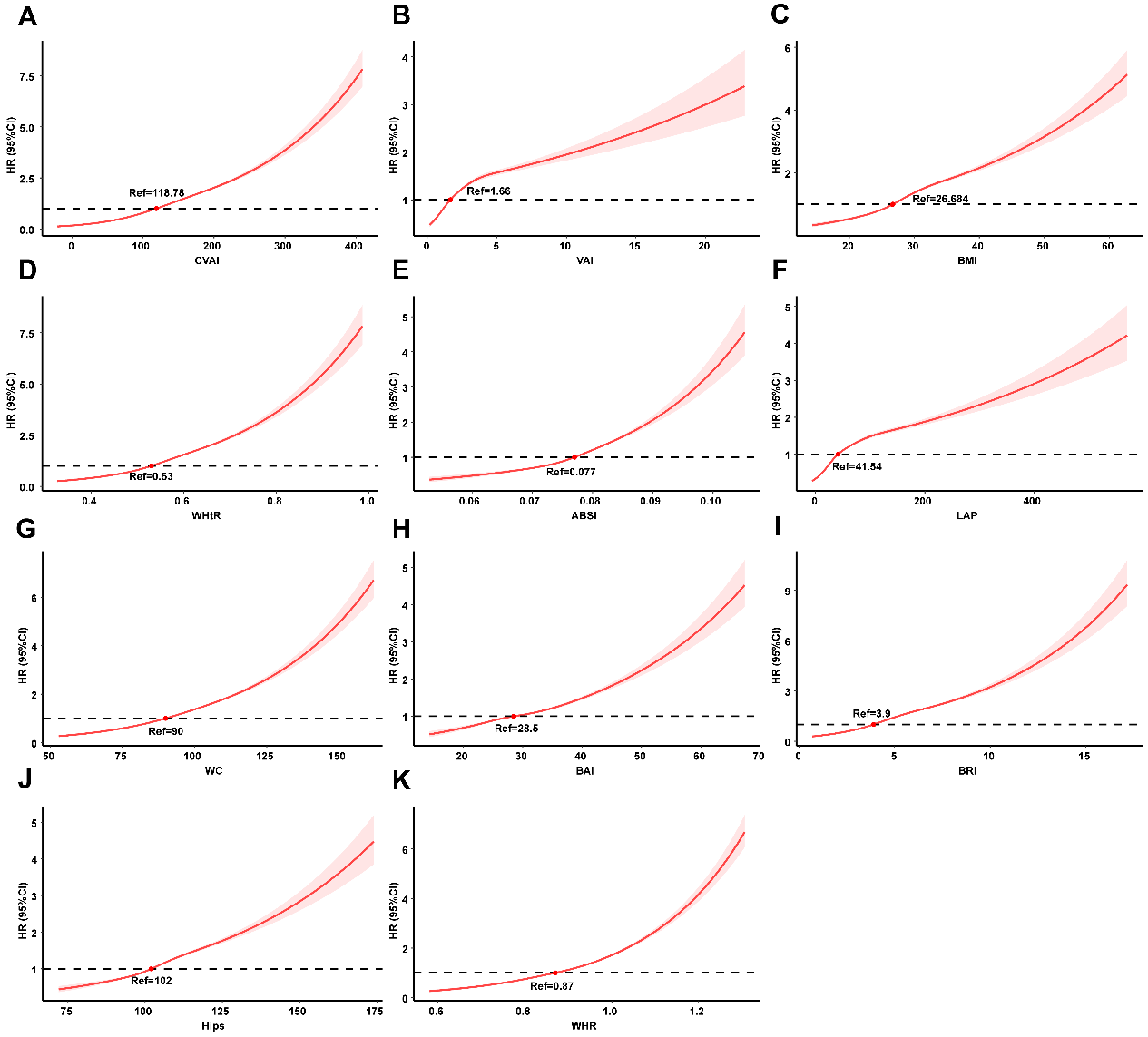


**Supplementary Figure 3.** Receiver operating characteristic curve in Binhai cohort.


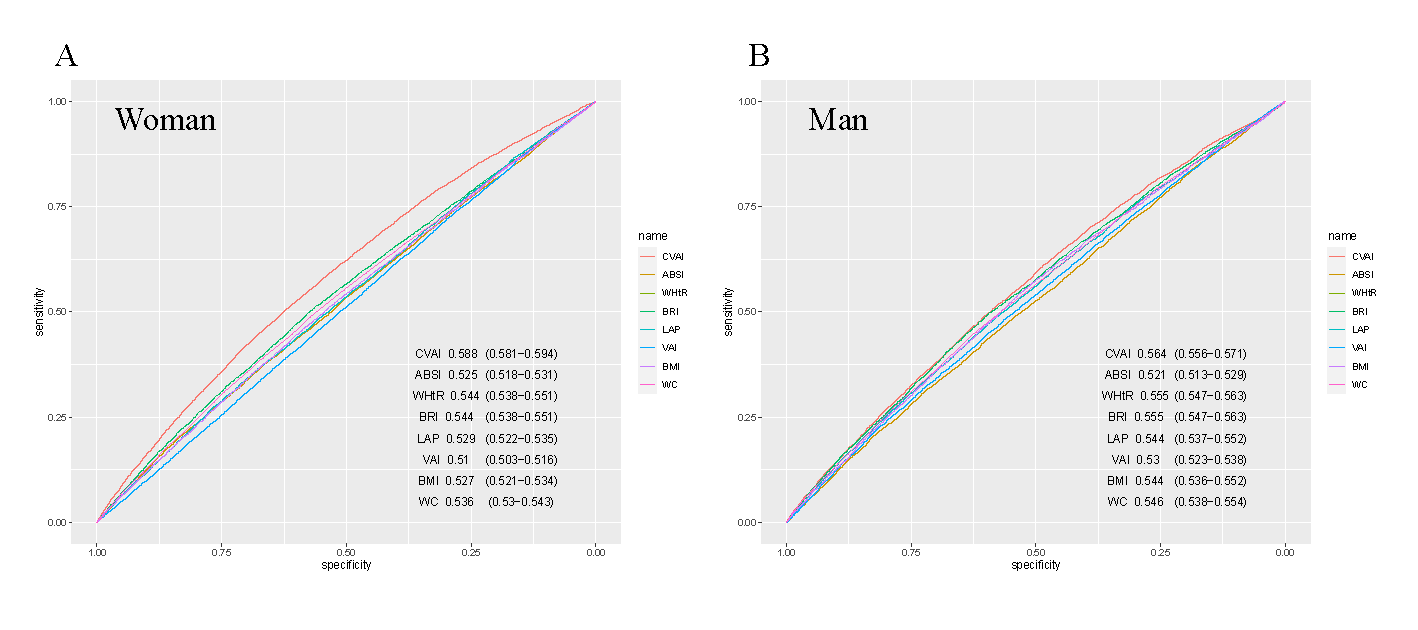


**Supplementary Figure 4.** Receiver operating characteristic curve in U.K. Biobank cohort.


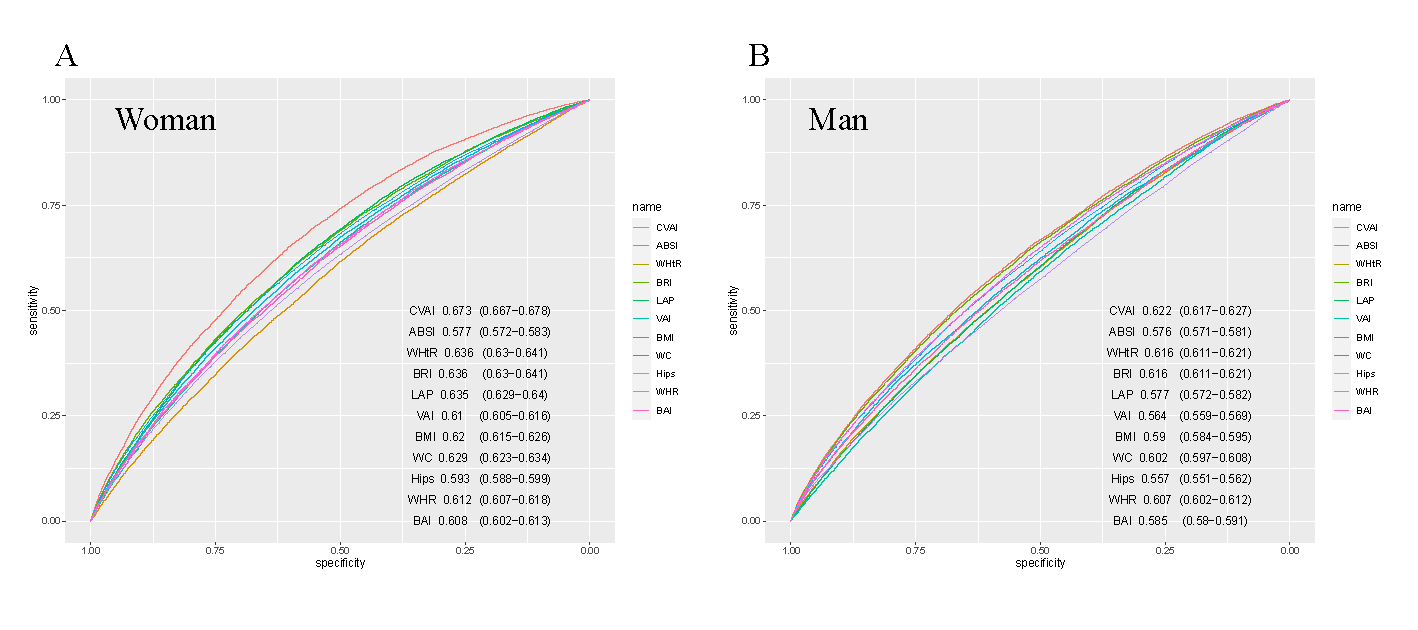


**Supplementary Figure 5.** The stratified analyses between obesity indexes and risk of CKD (U.K. Biobank cohort). (A) CVAI, Chinese visceral adiposity index. (B) BRI, body roundness index. (C) LAP, lipid accumulation product. (D) WC, waist circumference. (E) WHtR, waist-height ratio. (F) BMI, body mass index. (G) ABSI, a body shape index. (H) BAI, Body adiposity index. (I) Hips, Hip circumference. (J) VAI, visceral adiposity index. (K) WHR, Waist to hip ratio. Adjusted for age, gender, SBP, DBP, LDL-c, HDL-c, TC, TG, Cr at baseline, eGFR at baseline, smoking, drinking, history of diabetes, hypertension, coronary heart disease, cancer and stroke.


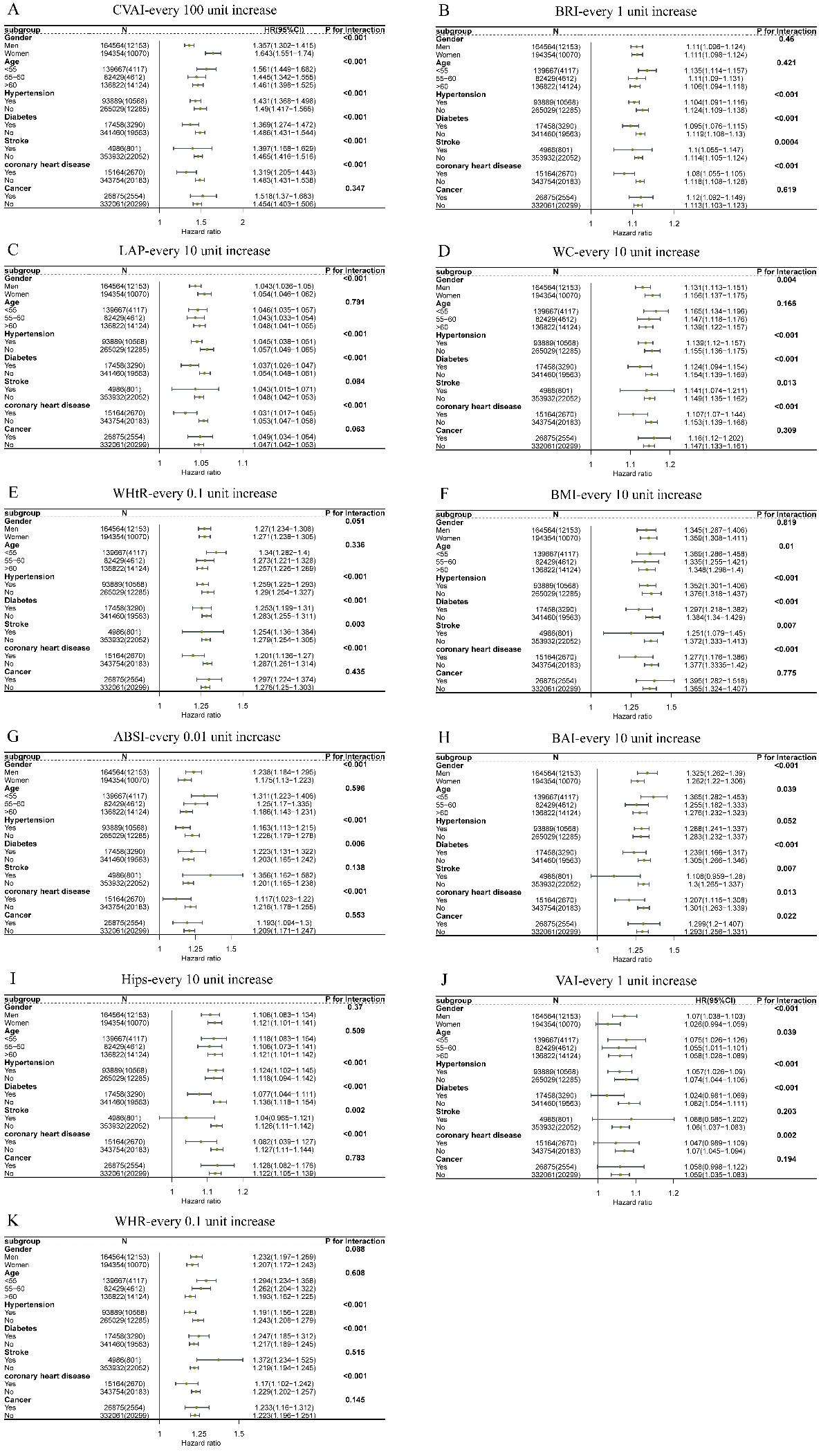

Supplement: Supplementary file 1 [file DataSheet1.docx]
